# Single‐Cell Sequencing Reveals That CCL2+ Adipose‐Derived Stem Cells Promote Diabetic Wound Healing Through the CCL2‐ACKR1 Signaling Axis

**DOI:** 10.1096/fj.202601311R

**Published:** 2026-04-25

**Authors:** Songyun Zhao, Wanying Chen, Kaibo Liu, Jiaheng Xie, Yanming Chen, Hao Dai, Zhongqiu Lu, Longwang Chen, Yucang He, Hua Yu, Liqun Li

**Affiliations:** ^1^ Department of Plastic Surgery The First Affiliated Hospital of Wenzhou Medical University Wenzhou China; ^2^ Cixi Biomedical Research Institute Wenzhou Medical University Cixi China; ^3^ Department of Plastic Surgery Xiangya Hospital Central South University Changsha China; ^4^ Department of Emergency Medicine The First Affiliated Hospital of Wenzhou Medical University Wenzhou China; ^5^ National Key Clinical Specialty (Wound Healing) The First Affiliated Hospital of Wenzhou Medical University Wenzhou China

**Keywords:** ACKR1, adipose‐derived stem cells, angiogenesis, CCL2, diabetic wound healing, exosomes, single‐cell RNA sequencing

## Abstract

Diabetic wounds, particularly diabetic foot ulcers, represent a significant clinical challenge owing to impaired vascularization, persistent inflammation, and dysfunctional extracellular matrix remodeling. Although adipose‐derived stem cells offer therapeutic potential, their heterogeneity and functional impairment within the diabetic microenvironment limit their efficacy. Using single‐cell RNA sequencing of human adipose and diabetic wound tissues, we identified a distinct CCL2‐expressing ADSC subpopulation that is enriched in obese individuals and exhibits elevated stemness, unique metabolic profiles, and enrichment in pathways related to ECM organization and tissue development. This subpopulation functions as a key communication node, engaging with fibroblasts, macrophages, and endothelial cells through ligand–receptor interactions such as CCL2‐ACKR1, TGFB1‐TGFBR1, and IL34‐CSF1R. Exosomes secreted by these CCL2‐positive ADSCs were found to be enriched in CCL2, TGFB1, and IL34. In a diabetic mouse wound model, CCL2‐ADSC‐derived exosomes significantly accelerated wound closure compared with conventional exosomes, promoting angiogenesis, collagen deposition, and M2‐macrophage polarization while reducing pro‐inflammatory cytokines. In vitro, these exosomes reversed high‐glucose‐induced suppression of endothelial cell proliferation, migration, and tube formation. Mechanistically, CCL2 carried by the exosomes activates the PI3K/AKT/mTOR/HIF‐1α signaling axis in endothelial cells via ACKR1, an effect abolished by CCL2 neutralization or ACKR1 knockdown. Together, these results demonstrate that the CCL2‐positive ADSC subpopulation exerts multi‐cellular and multi‐target therapeutic actions, and that exosomes derived from this subpopulation offer a potent cell‐free strategy to enhance diabetic wound healing by improving vascularization, modulating immune responses, and supporting ECM remodeling.

## Graphical abstract

scRNA‐seq of human adipose and diabetic wounds revealed a CCL2^+^ ADSC subpopulation with high stemness and ECM‐enriched pathways. These cells serve as a signaling hub via TGFB1‐TGFBR1, IL34‐CSF1R, and CCL2‐ACKR1. Their exosomes (CCL2‐Exos), carrying CCL2, TGFB1, and IL34, accelerate diabetic wound healing in mice by promoting angiogenesis, collagen deposition, M2 polarization, and reducing inflammation. Mechanistically, exosomal CCL2 activates endothelial PI3K/AKT/mTOR/HIF‐1α via ACKR1. CCL2‐Exos offer a multi‐target cell‐free therapy.

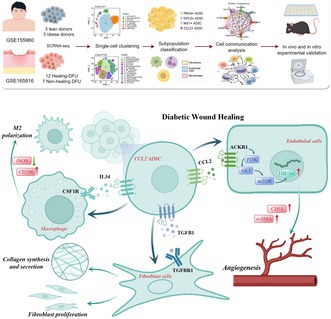

## 1 Introduction

Diabetes mellitus is a chronic metabolic disease frequently associated with multiple comorbidities. Among these, diabetic wounds, particularly diabetic foot ulcers (DFUs), present a common and significant therapeutic challenge in clinical practice [1]. Epidemiological data indicate that approximately 25% of individuals with diabetes are at risk of developing such wounds. This condition severely impairs the natural healing process, markedly increasing the patient's risk of chronic foot ulcers, lower‐limb amputation, and overall mortality [2, 3]. In China alone, 15%–25% of diabetic patients develop foot ulcers or gangrene, with associated treatment costs exceeding 700 billion RMB [4]. While current standard‐of‐care treatments—such as debridement, antibiotic administration, and revascularization—can effectively control infection in the short term, they have limited impact on the pathological microenvironment characteristic of chronic diabetic wounds, which includes persistent inflammation and impaired angiogenesis. This leads to high rates of recurrence [5]. The delayed healing in diabetic wounds is intricately linked to a complex pathophysiology hallmarked by compromised angiogenesis, a sustained inflammatory response, and suppressed immune function [6]. These factors not only prolong the healing timeline but also elevate the risk of infection and worsen clinical outcomes. Therefore, modulating inflammation and promoting angiogenesis have become critical intervention strategies aimed at breaking the vicious cycle of “treatment‐recurrence‐retreatment” [7].

Adipose‐derived mesenchymal stem cells (ADSCs) have emerged as a focal point in regenerative medicine due to their minimally invasive harvesting, low immunogenicity, and potent pro‐regenerative capabilities. A substantial body of research has shown that ADSCs secrete a variety of growth factors and cytokines that mediate angiogenesis, suppress inflammation, and promote tissue repair, demonstrating considerable promise for treating diabetic wounds [8]. However, the hyperglycemic and hypoxic microenvironment of these wounds significantly impairs ADSC function. To overcome this limitation, researchers have enhanced the paracrine activity and survival rate of ADSCs through the overexpression of HIF‐1α [9] or have improved cell viability, angiogenesis, and collagen deposition by loading the cells onto biomaterial scaffolds like poly‐L‐glutamic acid/chitosan [10]. More recently, ADSC‐derived exosomes (ADSC‐Exos)—which carry bioactive molecules such as miRNAs, proteins, and lipids—have been recognized as a cornerstone of “next‐generation cell‐free therapy.” These exosomes can alleviate oxidative stress and inflammation, thereby significantly promoting wound healing [11, 12]. To address the challenges of the short in vivo half‐life and poor targeting of free exosomes, strategies have been developed that combine hypoxia‐preconditioned exosomes with injectable hydrogels to achieve sustained release and enhanced pro‐angiogenic effects. Additionally, low‐intensity ultrasound stimulation has been shown to increase exosome secretion by an order of magnitude, substantially boosting their therapeutic potential [13]. Although early clinical data suggest that ADSCs and their exosomes have a favorable safety profile, the dynamic regulatory mechanisms by which the diabetic pathological microenvironment influences cell function still require in‐depth investigation [14, 15].

Currently, the therapeutic inconsistency of ADSCs, stemming from their inherent heterogeneity, remains a major obstacle to their clinical translation. The advent of single‐cell RNA sequencing (scRNA‐seq) provides a powerful tool to dissect the subset composition, functional characteristics, and environmental responses of ADSCs at single‐cell resolution. Within the diabetic wound, the synergistic effects of hyperglycemia, chronic inflammation, and hypoxia impair the survival, differentiation, and paracrine capacity of ADSCs, diminishing their pro‐healing efficacy [16]. Using scRNA‐seq, researchers can precisely identify distinct functional subsets, delineate their transcriptional signatures, and elucidate the underlying regulatory networks. This technology also enables the tracking of transplanted ADSC fate and the quantification of their interactions with host immune cells, revealing potential targets for optimizing therapeutic strategies [17]. Furthermore, integrating these findings with single‐cell atlases of the wound tissue itself can systematically elucidate the cellular and molecular basis of impaired healing, thereby pinpointing critical nodes for ADSC‐based intervention [18].

## 2 Materials and Methods

### 2.1 Download and Processing of Raw scRNA‐Seq Data

In this study, two single‐cell RNA sequencing (scRNA‐seq) datasets were obtained from the Gene Expression Omnibus (GEO) database for integrative analysis. The GSE165816 dataset [19] includes wound skin samples from 19 diabetic foot ulcer (DFU) patients (12 healing and 7 non‐healing cases). The GSE155960 dataset [20] contains CD45‐negative white adipose tissue data from 6 individuals (3 lean and 3 obese). The raw gene expression matrices were imported into the R environment using the Seurat package (v4). Data preprocessing strictly followed the steps outlined below to ensure data quality: The DoubletFinder package was applied to predict and remove potential doublets, thereby avoiding misclassification of cell types [21]; the DecontX package was used to correct for environmental RNA contamination [22]; stringent filtering criteria were applied to remove low‐quality cells: genes detected in 300–7000 counts, mitochondrial gene expression < 20%, and hemoglobin gene expression < 10% to exclude noise due to cell damage, stress, or erythrocyte contamination; the Harmony algorithm was used to correct for batch effects between samples, effectively removing technical variations while preserving biological differences [23]. Subsequently, data normalization was performed using the NormalizeData function, and the top 2000 most variable genes were selected as highly variable genes (HVGs) based on variance‐stabilizing transformation methods. The expression data were then scaled using the ScaleData function to remove potential sources of technical variation and further improve data quality. Cell clustering, as the core analysis step, was conducted as follows: Principal component analysis (PCA) was first performed on the selected HVGs, with the top 30 most informative principal components being retained; these principal components were then used for non‐linear dimensionality reduction via UMAP and t‐SNE to visualize the clustering structure; next, the FindNeighbors function (for constructing a shared nearest‐neighbor graph) and the FindClusters function (applying the Louvain algorithm) were used to identify cell clusters; finally, differential expression genes (DEGs) between clusters were identified using the FindAllMarkers function, and cell clusters were annotated by type and subtype based on the expression profiles of known canonical cell type markers. To further explore the heterogeneity within adipose‐derived stem cell (ADSC) subpopulations, we performed subclustering analysis on the ADSC population annotated from the primary clustering. The subcluster structure and its heterogeneity were visualized using t‐SNE dimensionality reduction.

### 2.2 Functional Enrichment Analysis

In this study, cell metabolic activity was assessed using the scMetabolism package [24]. For the enrichment analysis of single‐cell RNA sequencing data, we utilized the irGSEA package [25]. This tool integrates various gene ranking‐based enrichment scoring methods and enhances result robustness through a rank aggregation algorithm, providing multidimensional visualization capabilities. Additionally, we performed Gene Ontology (GO) and Kyoto Encyclopedia of Genes and Genomes (KEGG) enrichment analyses of differentially expressed genes (DEGs) using the clusterProfiler package [26] and conducted Gene Set Variation Analysis (GSVA) with the GSVA package [27]. The visualization of functional annotations was achieved through the ClusterGVis package, which integrates gene expression clustering, trend analysis, and enrichment results. It maps significantly enriched GO/KEGG pathways as color‐coded annotation blocks beside expression heatmaps, presenting an integrated display of expression patterns and functional annotations [28]. Cell type abundance was quantified using the Ro/e value (observed/expected ratio), representing the ratio of observed cell numbers to expected cell numbers for a specific cell type [29].

### 2.3 Trajectory Analysis and Stemness Inference

To assess the differentiation status and developmental potential of ADSCs, we performed CytoTRACE analysis on the scRNA‐seq data [30]. Based on the results of CytoTRACE and selecting normal skin cell populations as the starting point, we tracked the pseudo‐time trajectory of endothelial cells transitioning from the healthy group to the DFU group. Subsequently, the Monocle2 algorithm [31] was used to analyze the developmental trajectories of the inferred cell subpopulations. After dimensionality reduction and cell ordering, cell trajectories were inferred using the default parameters. Additionally, we employed the Slingshot package's getLineage and getCurves functions to infer the differentiation trajectories of each subpopulation [32].

### 2.4 Cell–Cell Communication and Transcription Factor Networks

To unravel the complex intercellular interaction networks within the wound microenvironment, we used the CellChat algorithm [33]. This computational method integrates transcriptomic profiles to reveal differential signaling modules between distinct cell populations. The analysis strictly followed the standard CellChat workflow, using the default CellChatDB as a reference database for ligand‐receptor interactions. By identifying cell‐type‐specific molecular features, such as the overexpression of signaling molecules, we inferred potential intercellular communication events. SCENIC, a tool for reconstructing gene regulatory networks (GRNs) and identifying stable cellular states based on scRNA‐seq data, was also employed. In this study, we utilized the PySCENIC package in Python [34] with default parameters for network reconstruction and transcription factor activity assessment.

### 2.5 Extraction of Adipose‐Derived Stem Cells (ADSCs) and Exosomes (Exos)

This study was approved by the Clinical Research Ethics Committee of the First Affiliated Hospital of Wenzhou Medical University (Ethical Review Number: KY2024‐R202). Detailed experimental procedures can be referenced from our previous studies on ADSCs [35, 36, 37, 38]. ADSCs were extracted from discarded adipose tissue obtained during liposuction surgery. The specific steps are as follows: The adipose tissue was centrifuged at 1200 rpm for 5 min, and the middle adipose layer was collected. This layer was then incubated with an equal volume of collagenase type I (Sigma, Cat. #9001‐12‐1) at 37°C for 1 h. After digestion, serum‐containing culture medium was added to inactivate the collagenase, and the solution was filtered through a 70 μm cell filter to obtain a single‐cell suspension. The suspension was centrifuged at 1000 rpm for 5 min to collect the cell pellet, which was then resuspended and seeded into culture dishes (cultured at 37°C with 5% CO₂). ADSCs were expanded using the basic culture medium (HUXMD‐90012) from Oricell (Guangzhou, China), and the medium was replaced every 3 days.

The identification of ADSCs was performed using flow cytometry (Beckman Coulter). The third‐generation cells were stained with PE‐conjugated antibodies (Abcam, anti‐human CD29/CD31/CD44/CD45 antibodies, 1:100 dilution) for surface marker analysis. CCL2^+^ADSC subpopulations were sorted using anti‐PDPN (Abcam, ab236529) and anti‐S1PR3 antibodies (Thermo, ASR‐013‐F‐50UL). Immunofluorescence staining of ADSCs followed previously described procedures, using an antibody targeting CCL2 (Thermo, MA5‐17040). The culture supernatant of ADSCs was collected, and CCL2, TGFB1, and IL34 activity were quantified using an ELISA kit (Abcam) according to the manufacturer's instructions.

All induction media for differentiation were purchased from Oricell (Guangzhou, China). Adipogenic Differentiation: A 1 mL volume of 0.1% gelatin solution was added to each well of a 6‐well plate and incubated at room temperature for 30 min to coat the surface, with excess gelatin removed. Once the cells reached 100% confluence, adipogenic induction medium A (HUXMD‐90031) was added and the cells were induced for 3 days. The medium was then replaced with adipogenic maintenance medium B (HUXMD‐90031) for 1 day. The induction (medium A) and maintenance (medium B) cycle was repeated. Cell morphology was observed daily, and once sufficiently sized lipid droplets appeared, Oil Red O staining was performed, and microscopic images were taken. Osteogenic Differentiation: The same gelatin coating procedure was used for the 6‐well plate. When the cells reached 70% confluence, osteogenic induction medium (HUXMD‐90021) was added, and the medium was changed every 3 days. After 2–4 weeks of induction, mineralization was evaluated by Alizarin Red staining to assess mineral nodule formation. Chondrogenic Differentiation: The protocol was similar to osteogenic differentiation, with the primary difference being the use of chondrogenic induction medium (HUXMD‐90041).

For exosome isolation, culture supernatant from the 3rd to 5th generation ADSCs was collected after 48 h of culture. The supernatant was centrifuged sequentially at 300 g, 2000 g, and 10 000 g for 30 min each (pellet discarded after each centrifugation) to remove cell debris and large particles. The supernatant was then subjected to ultracentrifugation at 120 000 g for 70 min, repeating this step twice. The final pellet was collected and resuspended in PBS to obtain the exosome suspension. The protein concentration of exosomes was quantified using the BCA method (Biyuntian), and the samples were stored at −80°C for further use. Characterization of the exosomes included transmission electron microscopy (TEM) to observe morphology, nanoparticle tracking analysis (NTA) to measure particle size distribution, and Western blotting to detect marker proteins (CD9, CD63, TSG101) and the negative control protein (Calnexin).

### 2.6 Monitoring of Wound Healing and Tissue Immunofluorescence in Mice

This study has been reported in line with the ARRIVE guidelines 2.0. One week prior to wound induction, 10‐week‐old male C57BL/6 mice were randomly selected for diabetes induction. After fasting for 10 h, the mice were intraperitoneally injected with streptozotocin (STZ, 50 mg/kg) for 5 consecutive days to induce type 1 diabetes. The success of the model was confirmed when fasting blood glucose levels exceeded 16.7 mmol/L, at which point continuous blood glucose monitoring was initiated. Mice were anesthetized with sodium pentobarbital (50 mg/kg), and the fur on their backs was shaved. The skin was disinfected with iodine solution, and a full‐thickness circular skin wound of approximately 1 cm in diameter was created on the back. Wound images were taken on postoperative Days 0, 3, 7, and 10. Wound area was measured using ImageJ software, and statistical analysis was performed. Blood flow to the wound was assessed using a laser Doppler blood flow imaging system. Mice were placed laterally on the imaging stage, adjusted to the optimal imaging plane, and blood flow was quantified using the MoorLDI ReviewV61 system, calculating the average blood flow within the wound area. On Day 10 after wounding, mice were deeply anesthetized with sodium pentobarbital (50 mg/kg, intraperitoneal injection) and subsequently euthanized by an overdose of sodium pentobarbital (150 mg/kg, intraperitoneal injection), followed by cardiac perfusion with phosphate‐buffered saline (PBS). Wound tissues were collected, fixed in formaldehyde, embedded in paraffin, and sectioned. After deparaffinization, hydration, and antigen retrieval, the sections were incubated with primary antibodies at 4°C overnight, followed by incubation with secondary antibodies at room temperature for 1 h. Finally, the sections were counterstained with DAPI to visualize nuclei, and images were captured using an inverted fluorescence microscope. Statistical analysis was performed using ImageJ software.

### 2.7 Cell Culture and Functional Assays

Human umbilical vein endothelial cells (HUVECs) were purchased from the Cell Bank of the Chinese Academy of Sciences. HUVECs were cultured in endothelial cell medium (Sciencell) at 37°C with 5% CO_2_. The glucose concentration in the medium was 5.5 mM for the normal glucose (NG) group and 30 mM for the high glucose (HG) group. siRNA transfection was carried out according to a previously described protocol, and cells were harvested 48 h post‐transfection for further experiments [39]. The sequence for the ACKR1‐targeted siRNA is as follows: ACKR1 siRNA, GCACGGAGCUGAAGGCUUU.

EdU incorporation was assessed using the BeyoClick Kit: when cells reached 60% confluence in 12‐well plates, they were incubated with 10 μM EdU labeling solution. After fixation with 4% paraformaldehyde for 30 min and permeabilization with 0.3% Triton X‐100 for 10 min, the click reaction was performed. The cells were counterstained with DAPI and proliferation was observed under a fluorescence microscope. The wound healing rate was determined using the scratch assay: after the cells were confluent, a vertical scratch was made with a 200 μL pipette tip with a ruler for guidance, and images were taken at set time intervals. Wound healing was quantified using ImageJ software. In the Transwell migration assay, 4 × 10^4^ cells in 100 μL serum‐free medium were placed in the upper chamber (8 μm pore size), while 500 μL of medium containing 20% serum was added to the lower chamber. After 24 h of incubation, non‐migrated cells were removed from the upper chamber, and the migrated cells in the lower chamber were fixed with 4% paraformaldehyde for 15 min, stained with crystal violet for 15 min, and washed with distilled water. The number of migrated cells was counted under a microscope. The tube formation assay was performed using GelNest matrix gel: 30 μL of gel was placed in each well of a 96‐well plate and solidified at 37°C for 30 min. Three thousand cells per well were seeded and cultured for 6 h, and tube‐like structures were observed under an inverted microscope. For more detailed experimental procedures, please refer to our previous publication [40].

Total RNA was extracted from HUVECs treated with different concentrations of CCL2‐Exos and Exos using TRIzol reagent according to the manufacturer's protocol. Subsequently, transcriptome sequencing was conducted by BGI (Shenzhen, China) on the DNBSEQ platform.

### 2.8 Western Blot

Total protein was extracted from each group using RIPA lysis buffer containing protease and phosphatase inhibitors. Protein concentration was quantified using a BCA protein assay kit. Equal amounts of protein (20–30 μg) were loaded onto an SDS‐PAGE gel for electrophoresis, followed by transfer to a PVDF membrane. The membrane was blocked with 5% skim milk in TBS‐T at room temperature for 1 h, then incubated overnight at 4°C with primary antibodies targeting the protein of interest. After washing with TBS‐T, the membrane was incubated with HRP‐conjugated secondary antibody at room temperature for 1 h. Protein bands were visualized using a chemiluminescent detection system, and quantification was performed with ImageJ software. GAPDH was used as a loading control for normalization. The following antibodies were used: CCL2 (Thermo, MA5‐17040); TGFB1 (Abcam, ab215715); IL34 (Abcam, 101 443); Calnexin (Abcam, ab92573); CD63 (Abcam, ab134045); TSG101 (Abcam, ab125011); CD9 (Abcam, ab236630); ACKR1 (Abcam, ab137044); PI3K (Abcam, ab191606); p‐PI3K (Abcam, ab278545); AKT (Abcam, ab8805); p‐AKT (Abcam, ab38449); mTOR (Abcam, ab2732); p‐mTOR (Abcam, ab109268); HIF‐1α (Abcam, ab1); and GAPDH (Abcam, ab8245). CCL2 neutralizing antibody (MAB679, R&D), human CCL2 recombinant protein (300‐04‐5, PeproTech).

### 2.9 Statistical Analysis

Statistical analysis was performed using R software (v4.2.1) and GraphPad Prism (v7.0). The methods included Student's *t*‐test, Wilcoxon test, or one‐way ANOVA. Results are presented as means ± standard deviation (SD). *p*‐values were adjusted using the false discovery rate (FDR) method, and all *p*‐values were two‐tailed. A *p*‐value < 0.05 was considered statistically significant.

## 3 Results

### 3.1 Single‐Cell Atlas Analysis of Adipose Tissue and Heterogeneity of Adipose‐Derived Stem Cells

**FIGURE 1:**
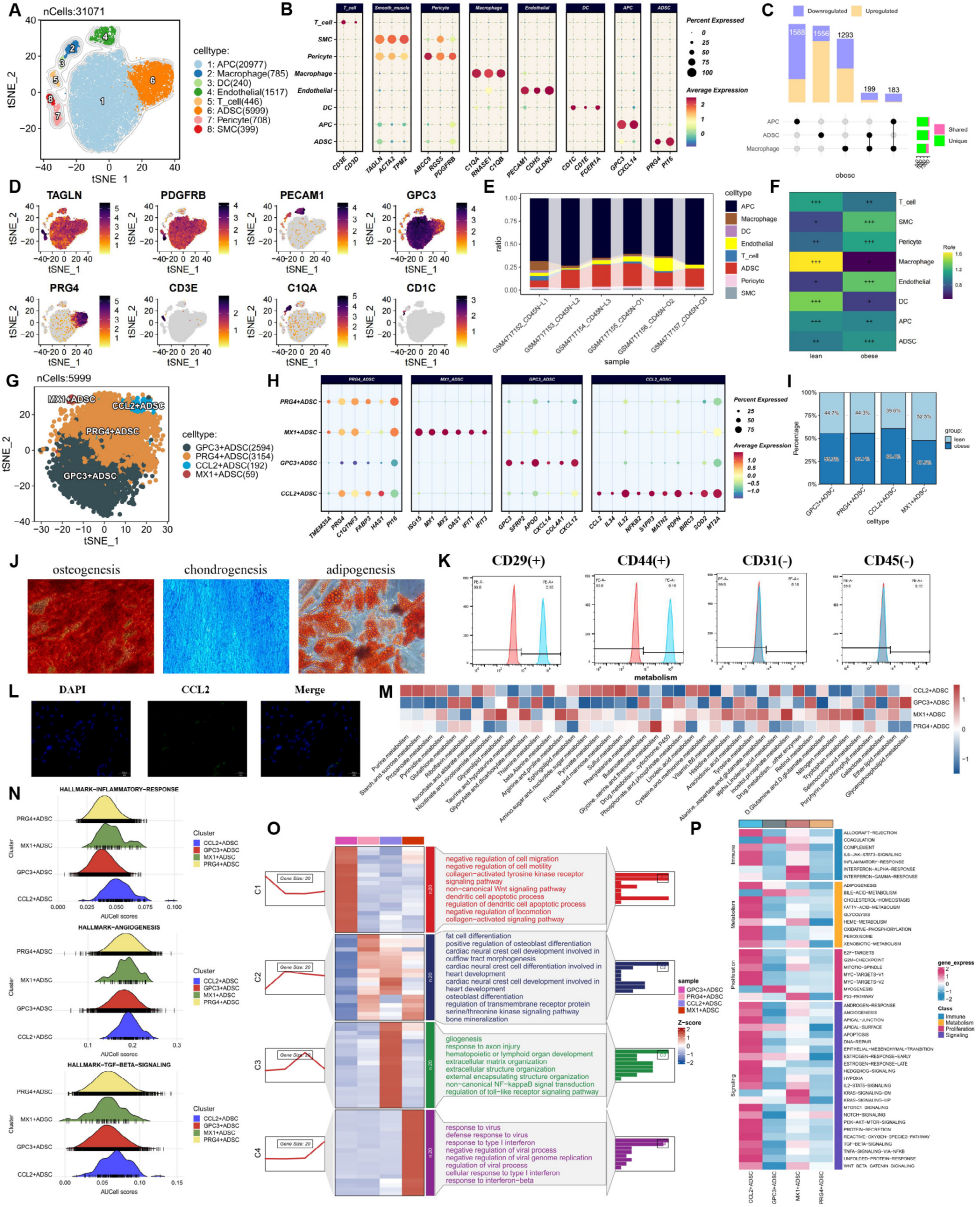
Single‐cell transcriptomic atlas of adipose‐derived stem cells. (A) t‐SNE dimensionality reduction visualization of the single‐cell atlas, color‐coded by eight major cell types. (B) Bubble plot showing the expression levels of characteristic marker genes across major cell types (red indicates high specific expression in the corresponding cell type). (C) Upset plot displaying the number of unique and shared differentially expressed genes (both upregulated and downregulated) between ADSC, APC, and macrophages. (D) t‐SNE plot showing the expression patterns of typical cell marker genes: Adipose‐derived stem cells (ADSCs) (PRG4), adipocyte precursor cells (APCs) (GPC3), pericytes (PDGFRB), smooth muscle cells (SMCs) (TAGLN), endothelial cells (PECAM1), T cells (CD3E), macrophages (C1QA), and dendritic cells (DCs) (CD1C). (E) Bar chart quantifying the relative distribution of each cell type across six samples. (F) Ro/e analysis assessing the enrichment or depletion status of cell subpopulations in different tissues (+++ indicates highly enriched, Ro/e > 1; ++ indicates significantly enriched, 0.8 < Ro/e ≤ 1; + indicates enriched, 0.2 ≤ Ro/e ≤ 0.8; +/− indicates weakly enriched, 0 < Ro/e < 0.2; − indicates no enrichment, Ro/e = 0). (G) t‐SNE plot showing the clustering of four adipose‐derived stem cell subpopulations. (H) Bubble plot showing the expression levels of characteristic marker genes across the four adipose‐derived stem cell subpopulations (red indicates high specific expression in the corresponding subpopulation). (I) Bar chart quantifying the relative distribution of the four adipose‐derived stem cell subpopulations. (J) Inverted differential interference contrast microscopy image showing the typical spindle‐shaped morphology of adipose‐derived stem cells, with the capacity to differentiate into adipocytes, osteocytes, and chondrocytes. (K) Flow cytometry confirming the expression of positive surface markers CD29 and CD44 in adipose‐derived stem cells, and the absence of negative surface markers CD31 and CD45. (L) Immunofluorescence confocal images of CCL2 expression in adipose‐derived stem cells (green). (M) Heatmap showing the metabolic pathway activities of the four adipose‐derived stem cell subpopulations. (N) Ridge plot showing the enrichment scores of specific pathways in the four adipose‐derived stem cell subpopulations. (O) Gene Ontology biological process (GO‐BP) enrichment analysis of the marker genes in the four adipose‐derived stem cell subpopulations. (P) Gene set variation analysis (GSVA) quantifying the hallmark signaling pathway activities in the four adipose‐derived stem cell subpopulations.

To investigate the immune microenvironment of adipose tissue and ADSC heterogeneity, we re‐analyzed publicly available single‐cell RNA sequencing (scRNA‐seq) data from six human adipose tissue samples deposited in the GEO database. After stringent quality control (including doublet removal and RNA contamination correction) and unsupervised clustering analysis, we identified cell clusters with similar expression patterns across the samples (Figure S1A–E). Referencing previous studies [40, 41, 42], we annotated major cell populations based on classical marker gene expression profiles: adipose‐derived stem cells (ADSCs, expressing PRG4 and PI16), adipocyte precursor cells (APCs, expressing GPC3 and CXCL14), pericytes (expressing ABCC9, RGS5, and PDGFRB), smooth muscle cells (SMCs, expressing TAGLN, TPM2, and ACTA2), endothelial cells (ECs, expressing PECAM1, CDH5, and CLDN5), T cells (expressing CD3D and CD3E), macrophages (expressing C1QA, RNASE1, and C1QB), and dendritic cells (DCs, expressing CD1C, CD1E, and FCER1A) (Figure 1A). Figure 1B shows a bubble plot illustrating the expression levels and distribution proportions of specific marker genes across major cell types, while t‐SNE dimensionality reduction visually presents the expression patterns of key marker genes across different cell types (Figure 1D). An Upset plot based on differential gene analysis quantified the number of unique and shared differentially expressed genes among ADSCs, APCs, and macrophages (Figure 1C). No significant differences were observed in the proportion or distribution preferences of ADSC and APC lineages between lean and obese samples (Figure 1E,F).

Given the multi‐mechanistic synergistic potential of adipose‐derived stem cells and their derivatives in addressing the limitations of traditional treatments for diabetic wound healing [43], we performed sub‐clustering analysis of ADSCs. Through t‐SNE dimensionality reduction, we identified four major subpopulations (Figure 1G). Using the FindAllMarkers function, we determined the highly expressed genes for each subpopulation (Table S1) and redefined them as follows: PRG4^+^ ADSCs were characterized by specific expression of genes associated with adipogenic differentiation and stem cell properties (e.g., PRG4, C1QTNF3, FABP3, and PI16), defining them as mature ADSCs; GPC3^+^ ADSCs were distinguished by unique expression of genes involved in lipid metabolism homeostasis and adipocyte precursor functions (e.g., GPC3, SFRP2, APOD, COL4A1, and CXCL14), representing a transitional state during ADSC differentiation into adipocyte precursor cells; MX1^+^ ADSCs were marked by specific expression of type I interferon‐stimulated genes (e.g., ISG15, MX1, MX2, OAS1, IFIT1, and IFIT3), suggesting a primary role in antiviral and inflammatory responses; CCL2^+^ ADSCs were enriched in genes related to immune regulation, antioxidant stress response, and extracellular matrix (ECM) remodeling (e.g., CCL2, IL34, IL32, NFKB2, S1PR3, MATN2, PDPN, BIRC3, SOD2, and MT2A) (Figure 1H). Notably, the proportion of CCL2^+^ ADSCs was elevated in obese samples (Figure 1I). To validate these findings, we isolated ADSCs and conducted a series of characterization experiments: phase‐contrast microscopy revealed their typical spindle‐shaped morphology; multi‐lineage differentiation assays confirmed their ability to differentiate into adipocytes, osteocytes, and chondrocytes (Figure 1J); flow cytometry demonstrated high expression of mesenchymal stem cell markers (CD29 and CD44) and low expression of hematopoietic markers (CD31 and CD45), consistent with their phenotypic profile (Figure 1K); immunofluorescence staining further confirmed CCL2 expression in a subset of ADSCs (Figure 1L). Given the metabolic heterogeneity among ADSC subpopulations, we systematically evaluated metabolic pathway activity using the scMetabolism method. CCL2^+^ ADSCs exhibited higher activity in glycolysis, lipid metabolism, and purine metabolism pathways (Figure 1M). Similarly, AUCell analysis revealed significantly elevated activity scores for CCL2^+^ ADSCs in inflammatory response, angiogenesis, and TGF‐β signaling pathways (Figure 1N). Gene Ontology (GO) enrichment analysis further delineated subpopulation‐specific functions: PRG4^+^ ADSCs were enriched in adipocyte differentiation pathways; GPC3^+^ ADSCs were associated with cell migration and motility; MX1^+^ ADSCs were primarily involved in antiviral defense responses; CCL2^+^ ADSCs were significantly enriched in tissue development and ECM‐related pathways (Figure 1O). HALLMARK pathway gene set variation analysis (GSVA) further supported that CCL2^+^ ADSCs displayed generally higher activity across multiple metabolism, immune, and proliferation‐related signaling pathways (Figure 1P). Collectively, these analyses demonstrate that the CCL2^+^ ADSC subpopulation exhibits a distinct gene expression profile and functional activity, positioning it as a key regulator in wound repair through synergistic modulation of inflammation, angiogenesis, and matrix remodeling. This underscores its potential as a promising therapeutic target for diabetic wound healing.

### 3.2 Differentiation and Transcriptional Regulatory Networks of Adipose‐Derived Stem Cells

**FIGURE 2:**
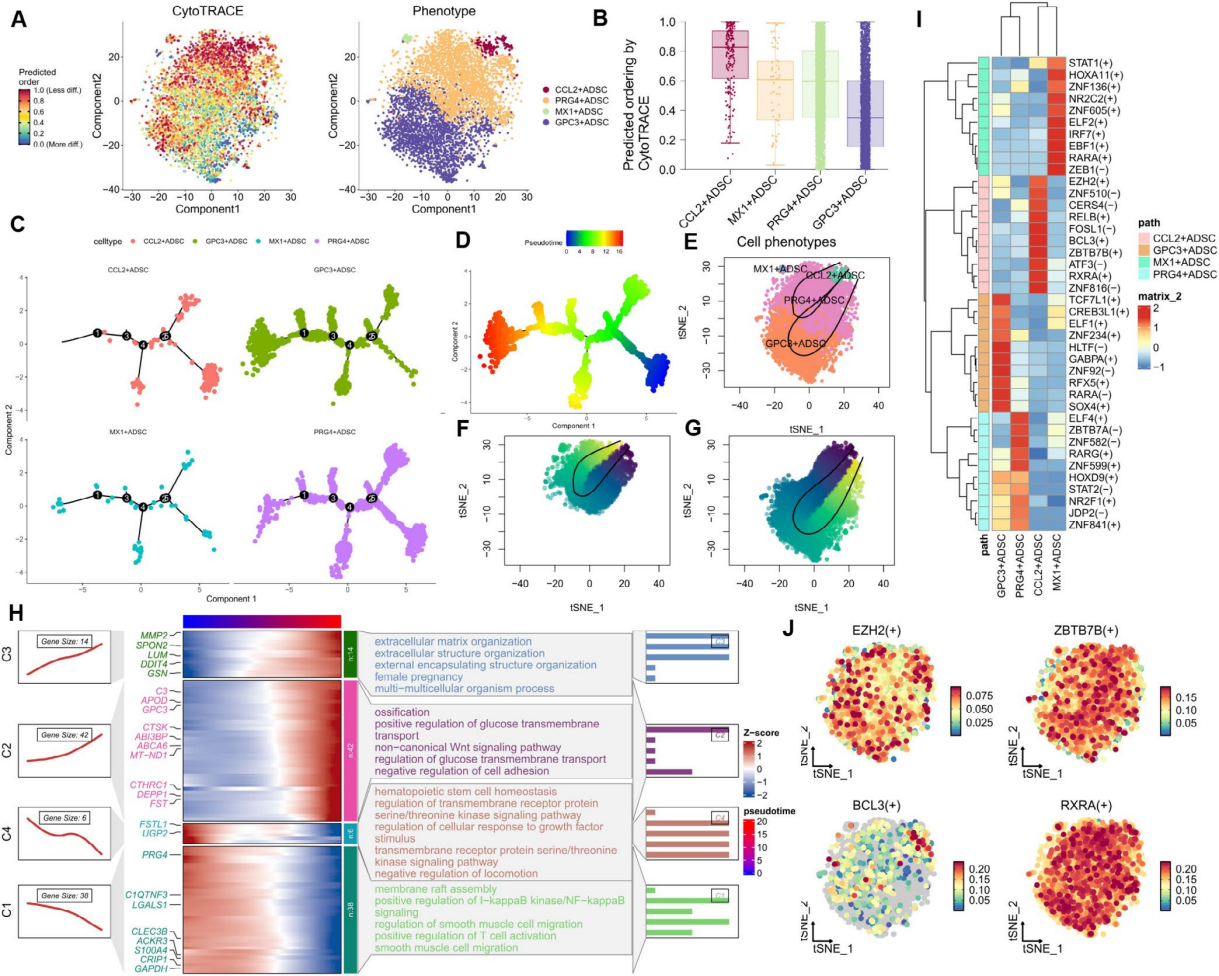
Differentiation trajectories of adipose‐derived stem cell subpopulations. (A, B) CytoTRACE analysis to assess stemness in the four ADSC subpopulations; higher CytoTRACE scores indicate increased stemness. Statistical significance was assessed using Kruskal‐Wallis test. CytoTRACE scores were mapped to the tSNE plot for each individual cell to visualize stemness variations across different adipose‐derived stem cell subpopulations. (C, D, H) Monocle2 pseudotime analysis showing the differentiation trajectories of the four ADSC subpopulations, with dynamic changes in GO‐BP enrichment results along the differentiation paths. (E, F, H) Slingshot analysis depicting two distinct differentiation paths in the ADSC development process. (I, J) Heatmap and tSNE plot showing the expression and activity of transcription factors (TFs) in the four ADSC subpopulations based on SCENIC analysis.

To assess the differentiation potential of adipose‐derived stem cell subpopulations, we utilized the CytoTRACE algorithm, which estimates cellular stemness (undifferentiated state) based on gene expression profiles. Higher stemness scores indicate greater differentiation potential. Our analysis revealed that the CCL2^+^ ADSC subpopulation exhibited the highest stemness score, suggesting a more primitive state with enhanced multilineage differentiation capacity. In contrast, the GPC3^+^ ADSC subpopulation showed the lowest stemness score, consistent with its role as a transitional state toward adipocyte precursor cell differentiation (Figure 2A,B). Monocle2 pseudotime analysis further demonstrated that CCL2^+^ ADSCs were predominantly enriched at the beginning of the differentiation trajectory (stemness maintenance state), while GPC3^+^ ADSCs clustered at the endpoint (terminal differentiation state) (Figure 2C,D). Notably, along the pseudotime axis, the activity of the NF‐κB inflammatory signaling pathway decreased progressively, while pathways involved in ECM remodeling showed sustained activation (Figure 2H). Slingshot pseudotime trajectory analysis identified two distinct differentiation paths: GPC3^+^ ADSCs and MX1^+^ ADSCs represented the two terminal states of the ADSC developmental trajectory (Figure 2E–G). Specifically, the GPC3^+^ endpoint was characterized by adipogenic differentiation, while the MX1^+^ endpoint signified an anti‐inflammatory/antiviral response state. This dual‐path differentiation model highlights the central role of CCL2^+^ ADSCs as a regulatory hub, directing the ADSC population toward a tissue repair phenotype by suppressing the NF‐κB inflammatory pathway and promoting ECM remodeling. Transcription factor prediction revealed that the regulatory activity of CCL2^+^ ADSCs was primarily linked to EZH2, ZBTB7B, BCL3, and RXRA (Figure 2I,J). A wound healing study demonstrated that EZH2 epigenetically silences the expression of transcriptional repressors ZNF24 and Runx1 via H3K27me3 modification. As ZNF24 and Runx1 act as transcriptional repressors of VEGFA, their downregulation results in increased VEGFA expression, which subsequently promotes endothelial cell proliferation, migration, and tube formation [44].

### 3.3 Single‐Cell Microenvironment Analysis of Diabetic Wound Tissue and Healing Signal Characteristics

**FIGURE 3:**
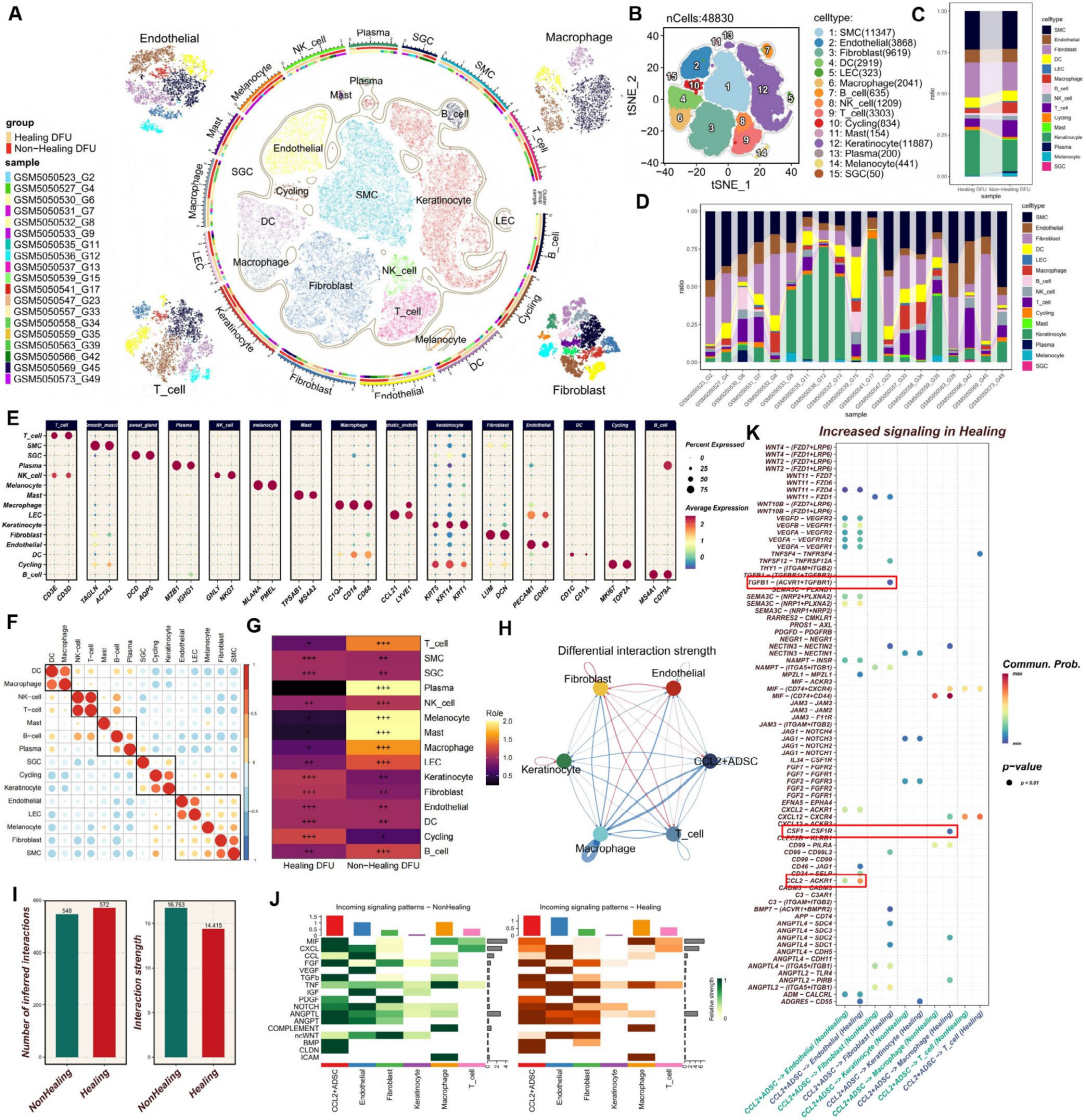
Single‐cell inflammatory microenvironment analysis of diabetic wound tissue and the pro‐healing signaling mechanisms of CCL2^+^ ADSCs. (A, B) t‐SNE dimensionality reduction visualization of the single‐cell atlas, color‐coded by eight major cell types, with sample information, source, and cell count. (C, D) Bar chart quantifying the relative distribution of each cell type across 19 samples (divided into two groups). (E) Bubble plot showing the expression levels of characteristic marker genes across major cell types (red indicates high specific expression in the corresponding cell type). (F) Correlation heatmap of single‐cell subpopulations. (G) Ro/e analysis evaluating the enrichment or depletion status of cell subpopulations in different tissues (Ro/e represents the ratio of observed to expected cell numbers; +++ indicates highly enriched, Ro/e > 1; ++ indicates significantly enriched, 0.8 < Ro/e ≤ 1; + indicates enriched, 0.2 ≤ Ro/e ≤ 0.8; +/− indicates weakly enriched, 0 < Ro/e < 0.2; − indicates no enrichment, Ro/e = 0). (H) Differences in the intensity of interactions between different cell populations. (Red: Upregulated in the healing group; Blue: Downregulated in the healing group). (I) Bar chart showing the differences in cell communication between healing and non‐healing groups based on the number and strength of interactions. (J) Comparison of signaling input strengths across all cell types in healing and non‐healing groups. (K) Identification of upregulated ligand‐receptor pairs in the healing group based on interaction probability comparison.

Using publicly available single‐cell RNA sequencing data, we conducted a comprehensive bioinformatic analysis of the inflammatory microenvironment in 19 diabetic wound tissue samples. After stringent quality control, including doublet removal and RNA contamination correction, and unsupervised clustering, we identified 25 immune and stromal cell subpopulations across the 19 samples (Figure S2A–E). By referencing prior studies [45, 46, 47] and classical marker gene expression profiles, we annotated the cell populations as follows: fibroblasts (DCN, LUM), endothelial cells (PECAM1, CDH5), smooth muscle cells (TAGLN, ACTA2), keratinocytes (KRT5, KRT14, KRT1), lymphatic endothelial cells (CCL21, LYVE1), sweat gland cells (DCD, AQP5), melanocytes (MLANA, PMEL), T cells (CD3E, CD3D), NK cells (GNLY, NKG7), macrophages (CSF1R, CD68, CD14), dendritic cells (CD1C, CD1A), mast cells (TPSAB1, MS4A2), B cells (MS4A1, CD79A), proliferating cells (MKI67, TOP2A), and plasma cells (MZB1, IGHG1) (Figure 3A,B). Comparative analysis revealed significant differences in the cell composition between healing and non‐healing wound samples (Figure 3C,D). Tissue preference analysis further revealed that non‐healing samples were enriched in immune cells, while healing samples had significantly higher proportions of fibroblasts and endothelial cells (Figure 3G). A bubble plot provided an overview of the expression levels and distribution proportions of specific marker genes across cell subpopulations (Figure 3E). Similarity clustering analysis based on marker gene expression profiles identified developmental and functional relationships among the different cell populations (Figure 3F).

Building on previous studies suggesting that CCL2^+^ ADSCs may promote diabetic wound healing, we further explored the reparative mechanisms between CCL2^+^ ADSCs and wound tissue cells. Specifically, this study focused on the ligand‐receptor signaling networks between CCL2^+^ ADSCs and key wound tissue cells, considering the dysregulated T cells and macrophages driving chronic inflammation, impaired endothelial cell function hindering angiogenesis, and dysfunctional fibroblasts leading to defective extracellular matrix deposition—all contributing to delayed wound healing. Cell communication analysis revealed that, compared to non‐healing tissues, healing tissues exhibited significantly stronger signaling interactions between CCL2^+^ ADSCs and fibroblasts (Figure 3H). Further quantification showed that healing samples had a broader cell communication network (with more interactions), while non‐healing samples exhibited higher individual interaction strengths (Figure 3I). Screening of wound healing‐related signaling pathways highlighted distinct signaling patterns between the healing and non‐healing groups (Figure 3J). Specifically, in healing wounds, three key signaling pathways showed significant activation: enhanced CCL2‐ACKR1 signaling between CCL2^+^ ADSCs and endothelial cells, upregulated TGFB1‐TGFBR1 signaling between CCL2^+^ ADSCs and fibroblasts, and increased CSF1‐CSF1R signaling between CCL2^+^ ADSCs and macrophages (Figure 3K).

### 3.4 Cell Communication Networks Involving CCL2 ^+^ ADSC in Wound Healing

**FIGURE 4:**
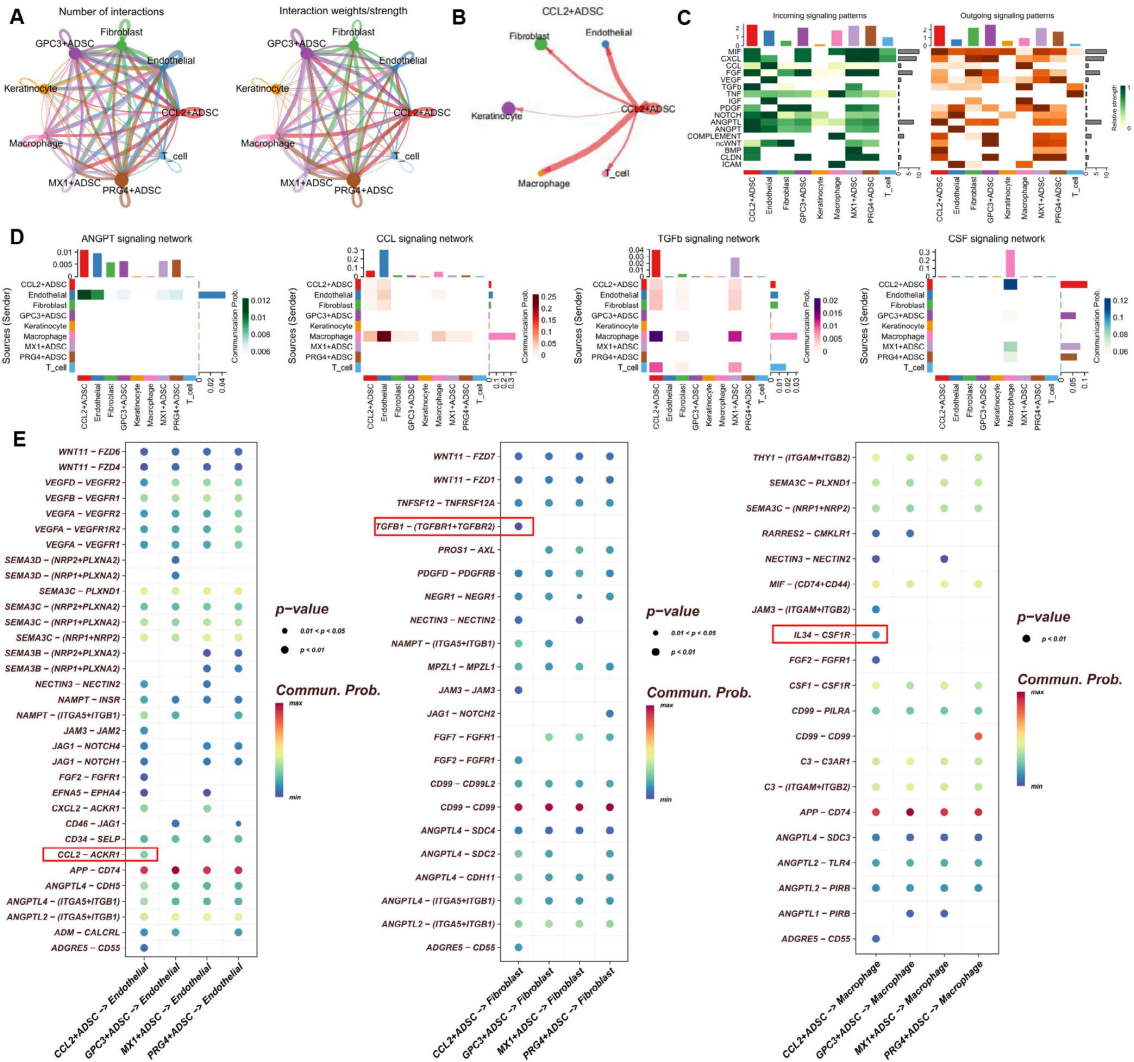
Cell communication networks of CCL2^+^ ADSCs in diabetic wounds. (A) Network visualization of interaction strength and quantity between the four ADSC subpopulations and other cell types. (B) Interaction strength between CCL2^+^ ADSCs and other cell types. (C) Heatmap showing the input and output strength of wound healing‐related signals across all cell types. (D) Heatmap showing the communication strength of four specific signaling pathways between different cell types. (E) Bubble plot showing key ligand‐receptor pairs involved in communication between CCL2^+^ ADSCs (signal sender) and fibroblasts, macrophages, and endothelial cells (signal receivers).

To validate our hypotheses, we performed a comprehensive cell communication analysis to evaluate the interaction networks between four ADSC subpopulations and cells within the inflammatory microenvironment. Figure 4A shows the distribution of interaction strength and quantity between the four ADSC subpopulations and other cell types. Notably, CCL2^+^ ADSCs emerged as a key interaction hub, particularly with fibroblasts, macrophages, and endothelial cells (Figure 4B). Further analysis revealed significant differences in the input/output patterns of wound healing‐related signaling pathways among CCL2^+^ ADSC, GPC3^+^ ADSC, PRG4^+^ ADSC, and MX1^+^ ADSC (Figure 4C). A heatmap quantified the interaction strengths of four key pathways—angiopoietin (ANGPT), chemokine (CCL), transforming growth factor‐β (TGF‐β), and colony‐stimulating factor (CSF)—across cell types (Figure 4D). Bubble plot analysis illustrated that CCL2^+^ ADSCs promote diabetic wound healing through three major therapeutic mechanisms: interactions with endothelial cells via the CCL2‐ACKR1 ligand‐receptor pair to facilitate pro‐angiogenic signaling; interactions with fibroblasts via TGFB1‐TGFBR signaling to promote collagen remodeling; and interactions with macrophages via pathways such as IL34‐CSF1R to mediate inflammation resolution. Importantly, the signaling strengths of these interactions were significantly higher in CCL2^+^ ADSCs compared to other ADSC subpopulations (Figure 4E).

### 3.5 CCL2 ^+^ ADSC‐Derived Exosomes (CCL2‐Exos) Accelerate In Vivo Wound Healing

**FIGURE 5:**
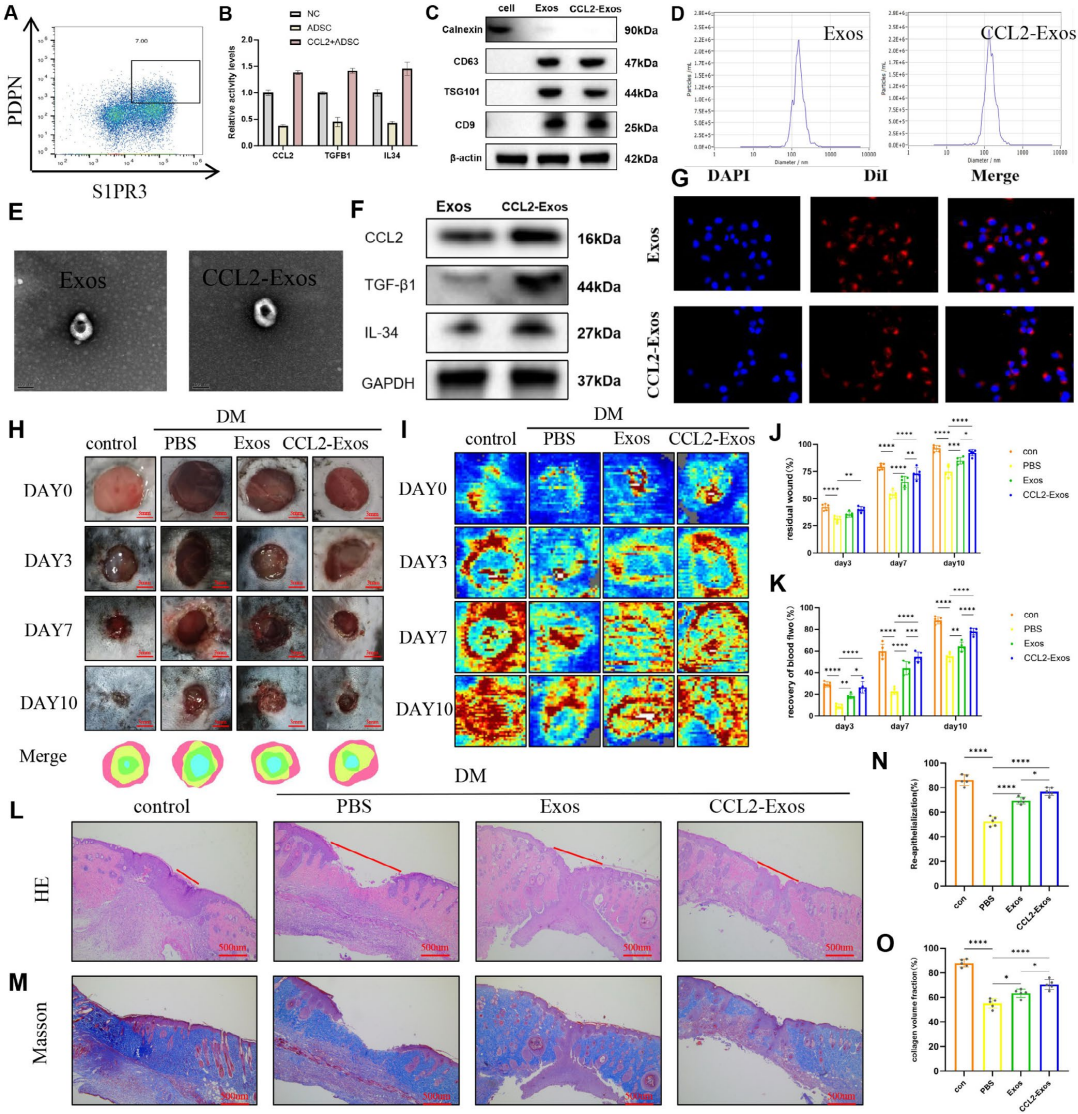
CCL2‐Exos promote in vivo wound healing. (A) Flow cytometry analysis to isolate PDPN^+^/S1PR3^+^ ADSCs (CCL2^+^ ADSCs) from adipose‐derived stem cells. (B) ELISA to evaluate the levels of CCL2, TGFB1, and IL34 in the culture supernatants of CCL2^+^ ADSCs. (C) Western blot analysis confirming the exosomal markers CD63, TSG101, and CD9, and the absence of calnexin, validating the exosome identity. (D) Nanoparticle tracking analysis (NTA) quantifying exosomes. (E) Transmission electron microscopy (TEM) providing high‐resolution images of Exos. (F) Western blot analysis identifying CCL2, TGFB1, and IL34 expression in Exos. (G) Fluorescent microscopy showing the internalization of DiI‐labeled Exos (red) by HUVECs. (H, J) Photos of the wound healing process in mice under different treatments, along with quantitative analysis of wound closure area. (I, K) Exos promoting angiogenesis, collagen deposition, and reducing inflammation. Laser Doppler imaging was used to capture wound blood flow, and corresponding quantitative analysis was provided. (L, N) Histological images of wound samples stained with H&E, and quantification of reepithelialization in the wound area. (M, O) Masson's trichrome staining of wound samples and quantitative analysis of collagen content in the wound area.

In our previous single‐cell RNA sequencing analysis, we observed significantly elevated expression of PDPN and S1PR3 in CCL2^+^ ADSCs (Table S1, Figure 1H). These markers enabled FACS isolation of the subpopulation. To further investigate the unique phenotype and functional role of CCL2^+^ ADSCs in wound healing, we employed fluorescence‐activated cell sorting (FACS) to isolate CCL2^+^ ADSCs based on their dual‐positive expression of PDPN and S1PR3 (Figure 5A). Enzyme‐linked immunosorbent assay (ELISA) results showed significantly higher concentrations of CCL2, TGFB1, and IL34 in the culture supernatants of CCL2^+^ ADSCs compared to regular ADSCs, which was consistent with our scRNA‐seq data (Figure 5B). We then isolated exosomes from the culture supernatants of regular ADSCs and CCL2^+^ ADSCs using ultracentrifugation, designating them as Exos and CCL2‐Exos, respectively. Western blot analysis confirmed the presence of characteristic exosomal markers (CD63, TSG101, and CD9) in both Exos and CCL2‐Exos, with no expression of the negative marker calnexin, confirming their identity as exosomes (Figure 5C). Nanoparticle tracking analysis (NTA) revealed that the particle size distribution of both Exos and CCL2‐Exos peaked at approximately 150 nm (Figure 5D), while transmission electron microscopy (TEM) showed the typical spherical or cup‐shaped vesicular morphology of both (Figure 5E). Notably, Western blot analysis further demonstrated that CCL2‐Exos had significantly higher levels of key secreted factors, including CCL2, TGFB1, and IL34, compared to Exos (Figure 5F). Using DiI fluorescent dye to label the exosomes, we observed efficient internalization of both Exos and CCL2‐Exos by human umbilical vein endothelial cells (HUVECs), confirming their potential for intercellular communication (Figure 5G).

To evaluate the efficacy of CCL2‐Exos in promoting diabetic wound healing in vivo, we established a streptozotocin (STZ)‐induced diabetic mouse model and created full‐thickness dorsal skin wounds in C57 mice. From Day 0 to Day 10 post‐surgery, wounds were subjected to different treatments. Macroscopic observations revealed that the CCL2‐Exos treatment group exhibited significantly greater wound contraction and faster healing compared to the regular ADSC‐derived exosome group (Figure 5H,J). Laser Doppler blood flow imaging analysis showed significantly higher blood flow signal intensity in the CCL2‐Exos group, indicating enhanced neovascularization and blood perfusion in the wound area (Figure 5I,K). On Day 10 post‐surgery, wound tissues were collected for histological analysis. Hematoxylin and eosin (H&E) staining indicated that the CCL2‐Exos group exhibited the shortest residual wound length and the most pronounced healing effect (Figure 5L,N). Masson's trichrome staining revealed denser collagen fiber arrangement and significantly increased collagen deposition in the CCL2‐Exos group, suggesting superior matrix remodeling (Figure 5M,O).

**FIGURE 6:**
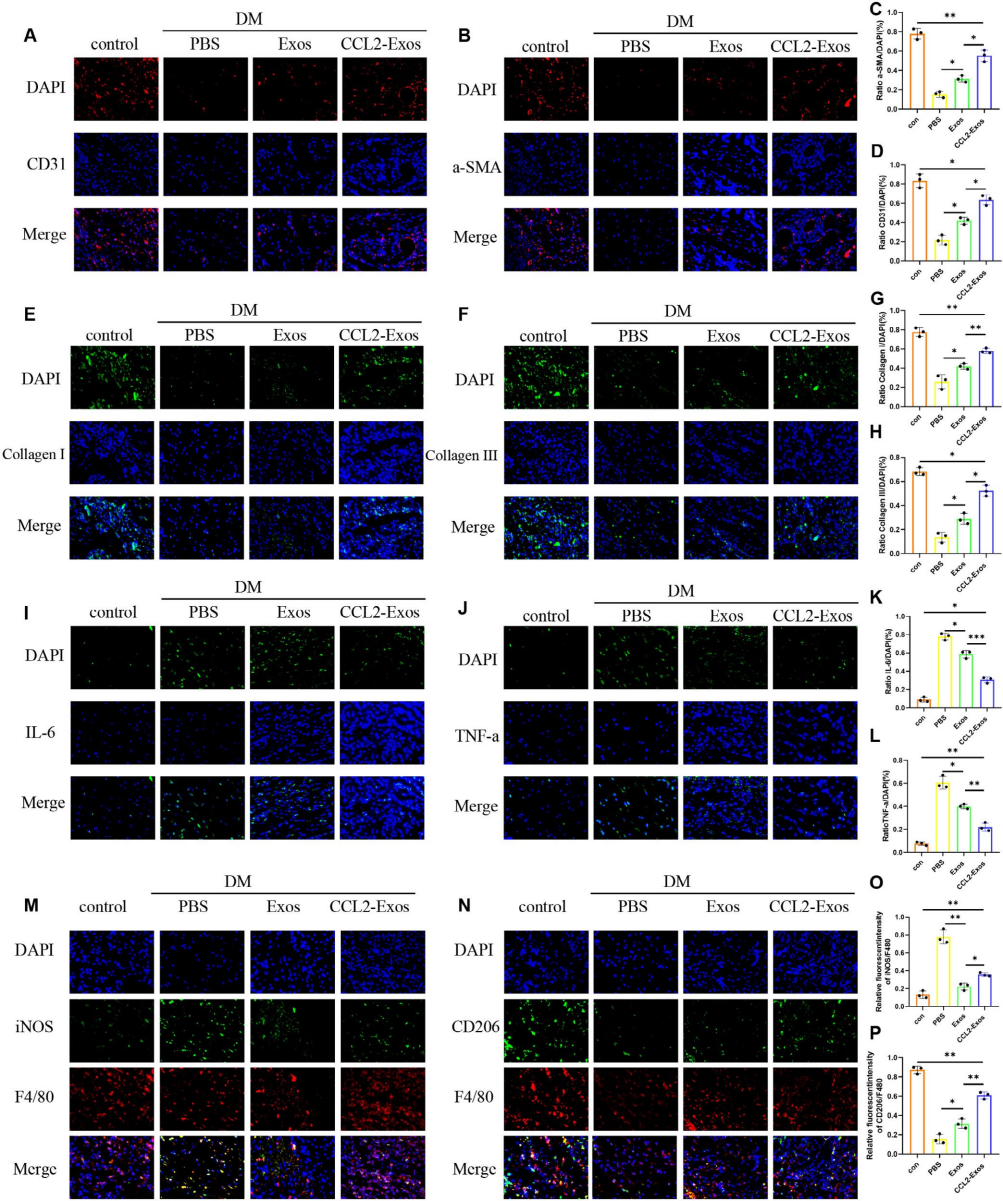
Immunofluorescence of wound tissue. (A–D) CD31 and α‐SMA representative images and quantification on day 10. (E–H) Collagen I and Collagen III representative images and quantification on day 10. (I–L) IL‐6 and TNF‐α representative images and quantification on day 10. (M–P) F4/80 and CD206 dual‐immunofluorescence staining on day 10 to indicate macrophage M2 polarization. Similarly, F4/80 and iNOS dual‐immunofluorescence staining on day 10 to indicate macrophage M1 polarization, followed by quantitative analysis.

Immunofluorescence analysis on Day 10 further elucidated the mechanisms by which CCL2‐Exos promote wound healing. In terms of angiogenesis, the CCL2‐Exos treatment group showed significantly upregulated expression of the vascular endothelial marker CD31 and the smooth muscle marker α‐SMA in wound tissue (Figure 6A–D). Collagen metabolism analysis revealed significantly increased deposition of type I and type III collagen in the CCL2‐Exos group (Figure 6E–H), indicating a more mature and stable extracellular matrix state. Regarding inflammation regulation, CCL2‐Exos significantly downregulated the expression of pro‐inflammatory cytokines IL‐6 and TNF‐α (Figure 6I–L). Macrophage phenotype analysis showed a significantly higher proportion of CD206^+^ (M2‐type) macrophages relative to F4/80+ (total) macrophages, and a significantly lower proportion of iNOS^+^ (M1‐type) macrophages in the CCL2‐Exos group (Figure 6M–P). These findings indicate that CCL2‐Exos effectively reshape the wound immune microenvironment by promoting macrophage polarization toward the reparative M2 phenotype while suppressing the pro‐inflammatory M1 phenotype. Collectively, these synergistic mechanisms significantly accelerate diabetic wound healing.

### 3.6 CCL2 ^+^ ADSC‐Derived Exosomes Regulate Endothelial Cell Proliferation and Migration via the CCL2‐ACKR1 Axis Under High‐Glucose Conditions

**FIGURE 7:**
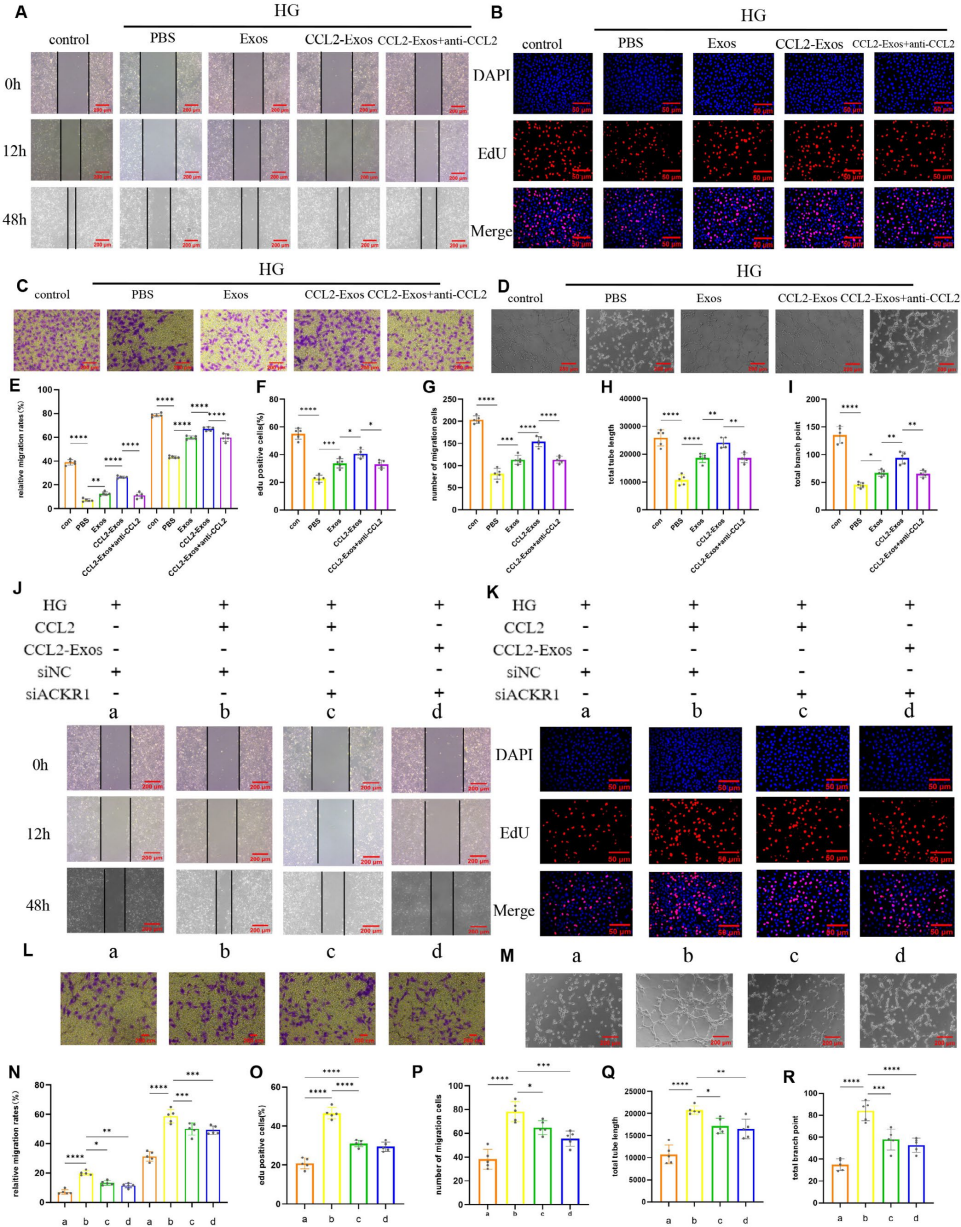
CCL2‐Exos promote angiogenesis, proliferation, and migration of HUVECs under high glucose conditions via the CCL2‐ACKR1 axis. (A, E) Scratch assay to evaluate the migration ability of HUVECs promoted by CCL2‐Exos, with quantitative analysis. (B, F) EdU incorporation assay to evaluate the DNA synthesis promoted by CCL2‐Exos in HUVECs. Red fluorescence represents EdU‐positive cells, and blue fluorescence represents total cells. Representative images and quantitative analysis of the EdU experiment. (C, G) Transwell assay to evaluate the migration ability of HUVECs promoted by CCL2‐Exos, with quantitative analysis. (D, H, I) Tube formation assay to evaluate the angiogenic potential of CCL2‐Exos, with quantitative analysis focusing on the total branch points and total tube length for each group. (J, N) Scratch assay to evaluate the migration ability of HUVECs regulated by the CCL2‐ACKR1 axis, with quantitative analysis. (K, O) EdU incorporation assay to evaluate DNA synthesis in HUVECs regulated by the CCL2‐ACKR1 axis. Red fluorescence represents EdU‐positive cells, and blue fluorescence represents total cells. Representative images and quantitative analysis of the EdU experiment. (L, P) Transwell assay to evaluate the migration ability of HUVECs regulated by the CCL2‐ACKR1 axis, with quantitative analysis. (M, Q, R) Tube formation assay to evaluate the angiogenic potential regulated by the CCL2‐ACKR1 axis, with quantitative analysis focusing on total branch points and total tube length for each group.

Scratch assay results demonstrated that CCL2‐Exosomes significantly mitigated high‐glucose‐induced damage in HUVECs compared to regular Exosomes (Figure 7A,E). EdU incorporation analysis revealed a significantly higher proportion of EdU‐positive cells in the CCL2‐Exos‐treated group under high‐glucose conditions (Figure 7B,F). Additionally, transwell migration assays confirmed that CCL2‐Exos notably enhanced cell migration in high‐glucose conditions (Figure 7C,G). Tube formation assays showed that CCL2‐Exos increased the number of branch points and total tube length under high‐glucose stimulation (Figure 7D,H,I). However, treatment with anti‐CCL2 neutralizing antibodies significantly reduced the proliferation‐promoting effects of CCL2‐Exos. Collectively, these results highlight that CCL2 within CCL2‐Exos stimulates cell proliferation, migration, and angiogenesis even under elevated glucose levels.

To validate the results of scRNA‐seq, CCL2 in CCL2‐Exos can directly interact with ECs through the ACKR1 receptor. Scratch assay results showed that after the addition of CCL2 recombinant protein treatment, HUVECs damaged by high glucose were significantly improved (Figure 7J,N). EdU analysis indicated that under high glucose conditions, the proportion of EdU‐positive cells significantly increased after treatment with CCL2 recombinant protein (Figure 7K,O). Additionally, Transwell migration assays demonstrated a significant enhancement in cell migration induced by CCL2 recombinant protein under high glucose conditions (Figure 7L,P). Tube formation assays further revealed that CCL2 recombinant protein under high glucose stimulation increased the number of branching points and total tube length (Figure 7M,Q,R). However, the knockdown of ACKR1 partially inhibited and reversed the CCL2‐Exos and CCL2 recombinant protein‐induced cell proliferation and migration in HUVECs.

**FIGURE 8:**
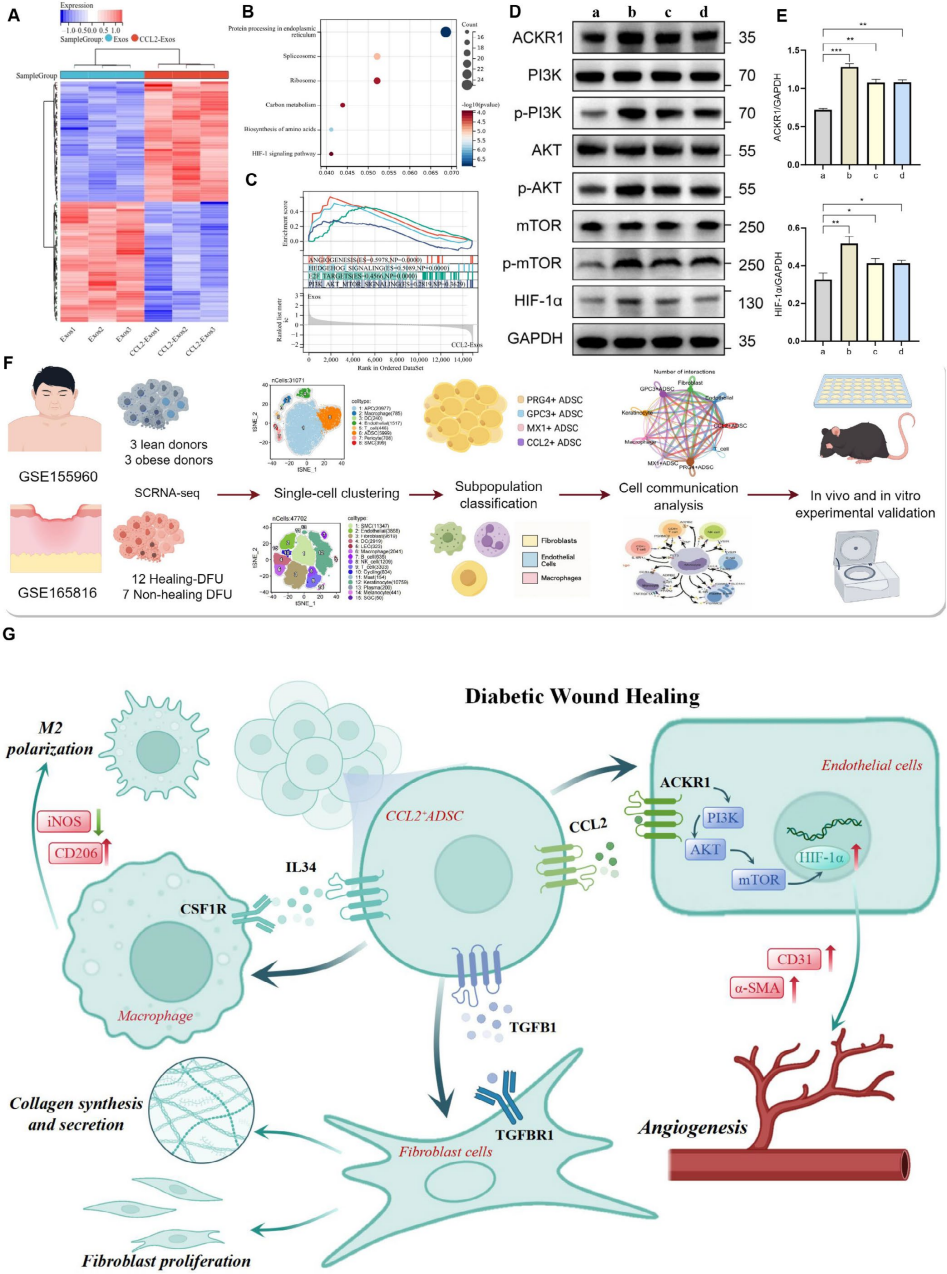
CCL2‐Exos promote angiogenesis through the ACKR1/PI3K/AKT/mTOR/HIF‐1α pathway. (A) Heatmap generated from transcriptomic sequencing data, showing upregulated and downregulated genes in the CCL2‐Exos group. (B) Bubble plot showing the KEGG pathway enrichment analysis results. (C) GSEA showing four signaling pathways upregulated in the CCL2‐Exos group. (D, E) Western blot analysis assessing the protein expression levels of ACKR1, PI3K, phosphorylated PI3K (p‐PI3K), AKT, phosphorylated AKT (p‐AKT), mTOR, phosphorylated mTOR (p‐mTOR), HIF‐1α, and GAPDH, along with quantitative analysis. (F) Flowchart summarizing the single‐cell sequencing and experimental validation processes of this study. (G) Diagram depicting the multicellular interaction mechanisms in diabetic wound repair, highlighting the molecular mechanisms by which CCL2^+^ ADSCs regulate target cell functions through three signaling pathways.

Heatmap analysis of transcriptomic sequencing data revealed differentially expressed genes in HUVECs treated with CCL2‐Exos compared to Exos (Figure 8A). KEGG enrichment analysis identified that these differentially expressed genes were enriched in several signaling pathways, including HIF‐1 (Figure 8B). Gene Set Enrichment Analysis (GSEA) further indicated upregulation of pathways related to angiogenesis, Hedgehog, and PI3K‐Akt signaling in HUVECs treated with CCL2‐Exos (Figure 8C). To investigate the potential mechanisms underlying CCL2‐Exos‐enhanced angiogenesis, we performed Western blot analysis to assess the expression levels of PI3K, AKT, mTOR, their phosphorylated forms, and HIF‐1α in HUVECs under high‐glucose conditions. The results showed that CCL2‐Exos and recombinant CCL2 protein significantly increased the phosphorylation of PI3K, AKT, and mTOR, along with enhanced expression of HIF‐1α. However, knockdown of ACKR1 partially suppressed CCL2‐induced activation of the PI3K/AKT signaling pathway in HUVECs (Figure 8D,E). Collectively, our single‐cell sequencing analysis revealed the therapeutic potential of CCL2^+^ adipose‐derived stem cells in diabetic wound healing. Further experimental validation confirmed that CCL2 produced by CCL2‐Exos activates ACKR1^+^ endothelial cells, thereby promoting angiogenesis through the PI3K/AKT/mTOR/HIF‐1α signaling pathway (Figure 8F,G).

## 4 Discussion

Adipose‐derived mesenchymal stem cells (ADSCs) hold great promise for the treatment of diabetic wounds, particularly diabetic foot ulcers (DFUs), owing to their convenient harvesting, abundance, excellent self‐renewal capacity, multipotent differentiation potential, and low immunogenicity [48]. Compared to mesenchymal stem cells (MSCs) from other sources, such as bone marrow or the umbilical cord, ADSCs offer distinct advantages in terms of minimal invasiveness of collection, higher yield, stronger proliferative capacity, and superior immunomodulatory activity, making them a preferred cell type for tissue repair and regenerative medicine. ADSCs accelerate the healing of chronic wounds by secreting a variety of cytokines, growth factors, and chemokines, which synergistically modulate the local immune microenvironment, promote neovascularization, and enhance collagen deposition. Mechanistically, ADSCs support neuroprotection and regeneration by secreting neurotrophic factors like BDNF, GDNF, bFGF, and IGF‐1 [49]. For angiogenesis, they upregulate pro‐angiogenic miRNAs and secrete factors such as VEGF, EGF, and FGF [50, 51]. This improves endothelial function and alleviates oxidative stress and inflammation through the activation of signaling pathways like PI3K/AKT/PTEN and mTOR‐HIF‐1α‐VEGF, as well as regulatory axes including lncRNA‐SENCR, SIRT3/SOD2, and Nrf2, ultimately mobilizing endothelial progenitor cells for neovascularization [52, 53, 54]. In terms of immunomodulation, ADSCs balance pro‐ and anti‐inflammatory responses, suppress the over‐activation of various immune cells, and promote macrophage polarization toward the M2 phenotype, thereby inhibiting NF‐κB signaling and mitigating chronic inflammation in the wound bed [55, 56]. Concurrently, ADSC‐derived exosomes (ADSC‐Exos), as a cell‐free therapy, have shown significant advantages by promoting the migration and proliferation of fibroblasts and keratinocytes, regulating ECM deposition and remodeling, and inhibiting scar‐associated proteins and signaling pathways to improve the quality of tissue healing [57, 58]. Despite the clear advantages of ADSCs in orchestrating tissue repair through multi‐target and multi‐pathway synergy, their clinical translation faces challenges. These include significant functional heterogeneity—as donor source, culture conditions, and passage number can all affect their biological properties and expression of pro‐reparative factors, leading to therapeutic variability [59, 60]. Furthermore, the unique pathological microenvironment of diabetic wounds, characterized by hyperglycemia, hypoxia, and chronic inflammation, further suppresses their survival, paracrine capacity, and differentiation potential [61].

To dissect the functional heterogeneity of ADSCs, this study utilized single‐cell RNA sequencing to identify a functional subcluster with a highly specific molecular signature within adipose tissue—the CCL2^+^ ADSC. This subcluster is characterized by high expression of genes such as CCL2, IL34, IL32, NFKB2, S1PR3, MATN2, PDPN, BIRC3, SOD2, and MT2A, and exhibits prominent immunomodulatory, antioxidant, and pro‐survival capabilities. Functional enrichment analysis revealed its significant enrichment in GO pathways related to tissue development and extracellular matrix remodeling. HALLMARK analysis showed it was active in core processes including metabolism, immune response, and cell proliferation. CytoTRACE analysis assigned this subcluster the highest stemness score, and Monocle2 trajectory analysis positioned it at the beginning of the differentiation path, indicating strong regenerative and differentiation potential. Notably, the proportion of CCL2^+^ ADSCs was significantly elevated in the adipose tissue of obese individuals, suggesting a close association with the metabolic stress environment, possibly representing a mechanism of passive expansion and active response under stress conditions. This finding challenges the traditional view of ADSCs as a homogeneous population, clarifies the unique role of this subcluster in immunomodulation, antioxidant protection, and tissue repair, and provides a new targeting direction for precisely selecting or enriching highly functional ADSC subclusters to enhance clinical efficacy.

Integrating our single‐cell transcriptomic data with existing literature, we systematically elucidated the multifaceted regulatory roles of CCL2 in wound healing, where it is particularly prominent in MSC‐mediated tissue repair. CCL2 is significantly upregulated in the early inflammatory phase of wound healing and can be produced by various cells (fibroblasts, endothelial cells, lymphocytes, and monocytes/macrophages) in an autocrine and paracrine manner [62]. It regulates inflammatory cell recruitment and neovascularization through both CCR2‐dependent and non‐canonical pathways. Inhibition of CCL2 prolongs healing time and reduces angiogenesis and epithelial proliferation, whereas its activation enhances VEGF expression and promotes vessel formation [63, 64, 65]. MSC‐secreted CCL2 is not only a key chemoattractant for monocyte–macrophage recruitment but also directly drives the polarization of M1 macrophages toward a reparative M2 phenotype, making it a critical molecule for maintaining immune homeostasis and transitioning from inflammation to repair [66, 67]. Moreover, the CCL2‐CCR2 signaling axis can directly induce the M2 phenotype; CCR2 deficiency significantly impairs IL‐10 secretion, while exogenous CCL2 can partially restore its function [68]. Upregulated IL‐10, in turn, can suppress neutrophil adhesion and infiltration, disrupting the inflammatory feedback loop and accelerating inflammation resolution [69]. In angiogenesis, MSC‐derived CCL2 promotes endothelial cell migration and proliferation, enhances HIF‐1 expression, and upregulates VEGF to drive neovascularization [70]. It also stimulates vascular smooth muscle cells to express tissue factor, promoting vessel maturation and functionalization, which provides a scaffold for epithelial cell migration and re‐epithelialization [71, 72]. The atypical chemokine receptor ACKR1, highly expressed at endothelial cell junctions, binds CCL2 with high affinity to regulate leukocyte adhesion and migration [73]. Hypoxia can induce high expression of CCL2 in epidermal cells [74, 75] and promote EMT and migration via the ERK1/2 signal, accelerating wound closure [76, 77]. Although ACKR1 is traditionally classified as an atypical chemokine receptor lacking classical G‐protein signaling, emerging evidence indicates that ACKR1 can modulate endothelial activation through chemokine presentation, spatial confinement, and non‐canonical signaling mechanisms. In the diabetic wound microenvironment, where endothelial cells are highly responsive to chemokine cues, ACKR1 may function as a signaling scaffold that facilitates CCL2‐induced activation of the PI3K/AKT/mTOR pathway. Our findings that ACKR1 knockdown markedly attenuated CCL2‐mediated angiogenic responses support this context‐dependent signaling role of ACKR1. Unlike typical chemokine receptors such as CCR2, ACKR1 does not induce Gαi‐dependent calcium flux or chemotaxis. Instead, its high expression on endothelial junctions positions it to act as a signaling scaffold. We propose that CCL2 binding to ACKR1 may recruit β‐arrestin or other adaptors, thereby scaffolding PI3K to the membrane and activating the AKT/mTOR/HIF‐1α axis. Alternatively, ACKR1 may concentrate CCL2 locally to facilitate engagement of a distinct co‐receptor that directly transduces the signal. While our loss‐of‐function data support this model, the exact molecular interactions remain unresolved. Future studies using proximity‐based proteomics and receptor mutagenesis are required to confirm whether ACKR1 directly interacts with PI3K or operates through an intermediary.

Our study reveals that CCL2^+^ ADSCs not only possess immunomodulatory, antioxidant, and pro‐reparative advantages but also act as a signaling hub in wound repair. They establish a multi‐nodal regulatory network across different cell types through high‐intensity interactions with fibroblasts, macrophages, and endothelial cells. The CCL2‐ACKR1 axis targets endothelial cells, activating the PI3K/AKT/mTOR/HIF‐1α pathway to enhance their proliferation, migration, and tube formation, thereby improving the insufficient blood supply in DFUs. The TGFB1‐TGFBR1 pathway regulates collagen synthesis and matrix remodeling by fibroblasts, while the IL34‐CSF1R signal promotes M2 polarization and suppresses inflammatory responses. This multi‐pathway synergistic mechanism breaks the vicious cycle of “vascular impairment—persistent inflammation—matrix imbalance” in diabetic wounds, providing a theoretical and practical basis for improving the quality of chronic wound repair. It also suggests that enriching or activating CCL2^+^ ADSCs could be a novel strategy for optimizing regenerative therapies.

To overcome the potential risks of ADSC therapy, such as immune rejection, tumorigenicity, and ethical constraints, ADSC‐derived extracellular vesicles (ADSC‐EVs) are emerging as a focal point for cell‐free therapies. ADSC‐EVs retain many of the activities of their parent cells—including immunomodulation, anti‐apoptosis, pro‐angiogenesis, and neuroregeneration—while offering higher in vivo stability and lower immunogenicity. They can efficiently deliver proteins and nucleic acids to damaged tissues, achieving comparable or even superior therapeutic effects while reducing the risks of direct cell transplantation [78, 79]. This study further demonstrated that exosomes derived from CCL2^+^ ADSCs are enriched with regulatory factors like CCL2, TGFB1, and IL34. While our study focuses on CCL2, it is important to note that CCL2‐Exos also carry high levels of TGFB1 and IL34. TGFB1 promotes fibroblast‐mediated collagen remodeling, and IL34 supports M2 macrophage polarization. Thus, the pro‐healing effects of CCL2‐Exos likely arise from synergistic actions among these factors. Nevertheless, our neutralization experiments demonstrate that CCL2 is indispensable, as its inhibition largely abrogates the exosomal benefits. Future studies should dissect the relative contributions of TGFB1 and IL34 and their crosstalk with CCL2. These exosomes were efficiently internalized by HUVECs, significantly accelerated wound closure in diabetic mice, improved blood perfusion, enhanced neovascularization and collagen deposition, promoted M2 polarization, and downregulated inflammatory factors. In vitro, CCL2‐Exos reversed the high‐glucose‐induced suppression of endothelial cell proliferation, migration, and tube formation. Mechanistic studies showed that their pro‐reparative effects are dependent on the CCL2‐ACKR1 signaling axis, as ACKR1 knockdown or a CCL2 neutralizing antibody significantly attenuated these effects. RNA‐seq and GSEA analyses also confirmed that CCL2‐Exos upregulated genes associated with angiogenesis and the PI3K‐AKT pathway.

Multiple scRNA‐seq predictions were experimentally validated in this study: (i) a distinct CCL2^+^ ADSC subpopulation with high stemness and ECM‐related pathways (CytoTRACE, pseudotime, differentiation assays); (ii) elevated CCL2, TGFB1, and IL34 expression (ELISA/Western blot); (iii) key ligand‐receptor interactions CCL2‐ACKR1, TGFB1‐TGFBR1, and IL34‐CSF1R (neutralizing antibodies, knockdown, recombinant proteins); (iv) pro‐angiogenic signaling via PI3K/AKT/mTOR/HIF‐1α downstream of ACKR1 (Western blot, tube formation). Unvalidated predictions, including transcription factor networks (EZH2, RXRA), remain for future investigation. In summary, the therapeutic effects of CCL2^+^ ADSCs are at least partially mediated by their exosomes, with the CCL2‐ACKR1 axis playing a decisive role in angiogenesis within diabetic wounds. CCL2‐Exos stand out as a multi‐potent, cell‐free therapeutic vehicle, offering a novel theoretical basis and pathway for developing more targeted and controllable regenerative strategies. This research not only provides new insights into the mechanisms underlying ADSC therapeutic variability and avenues for clinical optimization but also highlights the potential therapeutic value of ACKR1. While limitations exist—such as differences in model applicability, unoptimized delivery strategies and dosages, and the unelucidated mechanisms of other active components—future research should focus on efficiently enriching human CCL2^+^ ADSCs, optimizing exosome engineering and delivery systems, elucidating the synergistic mechanisms of other key active factors, and developing ACKR1 agonists or combination therapies. Advancing pre‐clinical studies of CCL2‐Exos for clinical indications like DFUs will be crucial for accelerating their clinical translation.

## Author Contributions

Songyun Zhao and Wanying Chen conceived and designed the study and performed the experimental work. Hua Yu, Kaibo Liu, Hao Dai, Jiaheng Xie, Zhongqiu Lu, Longwang Chen, and Yanming Chen provided supervision and contributed to data interpretation. Songyun Zhao wrote the initial draft of the manuscript, which was critically revised for intellectual content by Yucang He and Liqun Li. All authors reviewed and approved the final version of the manuscript.

## Funding

This study was supported by the Zhejiang Clinovation Pride (CXTD202501043).

## Ethics Statement

All animal experimental protocols were approved by the Ethics Committee of the First Affiliated Hospital of Wenzhou Medical University [Approval title: Adipose mesenchymal stem cells‐hydrogels promote wound healing; Approval number: WYYY‐IACUC‐AEC‐2025‐021] (Date of approval: February 2025). The protocol for collecting adipose‐derived stem cells was approved by the Ethics Committee of the First Affiliated Hospital of Wenzhou Medical University [Approval title: Adipose mesenchymal stem cells‐hydrogels promote wound healing; Approval number: KY2024‐R202] (Approval period: December 2024). The study was conducted in accordance with local legal and institutional requirements.

## Consent

Written informed consent was obtained from all patients (or their guardians/legally authorized representatives) for participation in the study and for the use of their adipose tissue samples. The human umbilical vein endothelial cells (HUVECs) used in this study were purchased from the Cell Bank of the Chinese Academy of Sciences (Shanghai, China). According to the provider's official statement, the cell bank obtained the cells under ethical approval, and the donors had provided written informed consent at the time of sample collection. All experiments using HUVECs were conducted in compliance with institutional and national ethical standards.

## Conflicts of Interest

The authors declare no conflicts of interest.

## Supporting information


**Figure S1:** Through stringent quality control and unsupervised clustering analysis, cell clusters with similar expression patterns across adipose tissue samples were identified.
**Figure S2:** Through stringent quality control and unsupervised clustering analysis, cell clusters with similar expression patterns across diabetic wound samples were identified.
**Table S1:** Highly expressed genes in various subpopulations of adipose‐derived stem cells were screened using the FindAllMarkers function.

## Data Availability

Publicly available datasets were analyzed in this study. The raw data for this study were obtained from GEO (http://www.ncbi.nlm.nih.gov/geo/) databases.
