# Supplementary material for: Single‐Cell Sequencing Reveals That CCL2+ Adipose‐Derived Stem Cells Promote Diabetic Wound Healing Through the CCL2‐ACKR1 Signaling Axis
Source: FASEB J. 2026 Apr 25;40:e71853. doi: 10.1096/fj.202601311R (PMC13109809; doi:10.1096/fj.202601311R)
Supplement: Supplementary file 1 — Figure S1: Through stringent quality control and unsupervised clustering analysis, cell clusters with similar expression patterns across adipose tissue samples were identified. Figure S2: Through stringent quality control and unsupervised clustering analysis, cell clusters with similar expression patterns across diabetic wound samples were identified. Table S1: Highly expressed genes in various subpopulations of adipose‐derived stem cells were screened using the FindAllMarkers function. [file FSB2-40-e71853-s001.pdf]

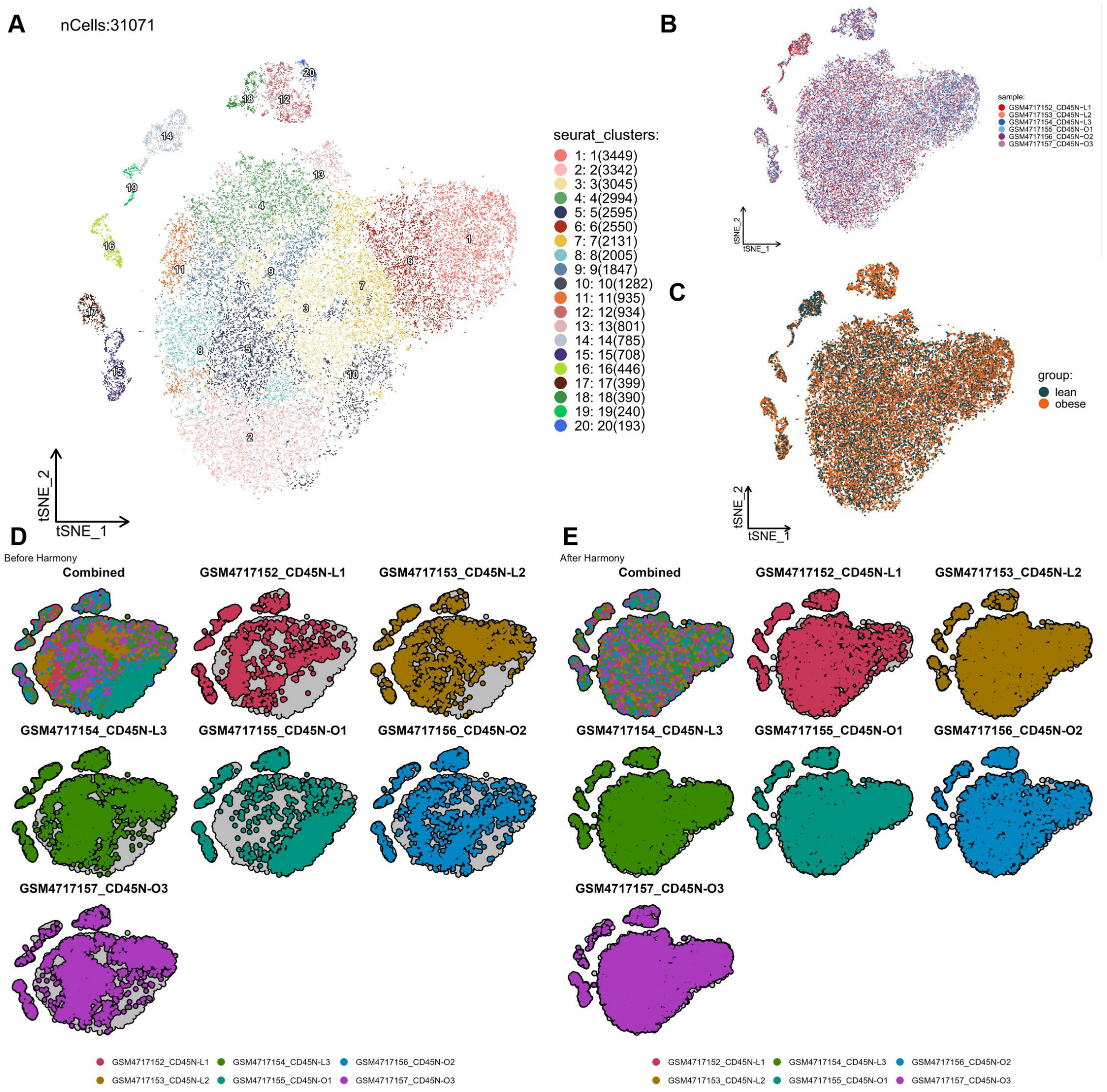

Supplementary Figure 1. *Through stringent quality control and unsupervised clustering analysis, cell clusters with similar expression patterns across adipose tissue samples were identified. (A-C) t-SNE visualization showing the distribution of cells by cluster, sample, and tissue origin. (D,E) t-SNE distribution of cells before and after batch effect removal using Harmony.*

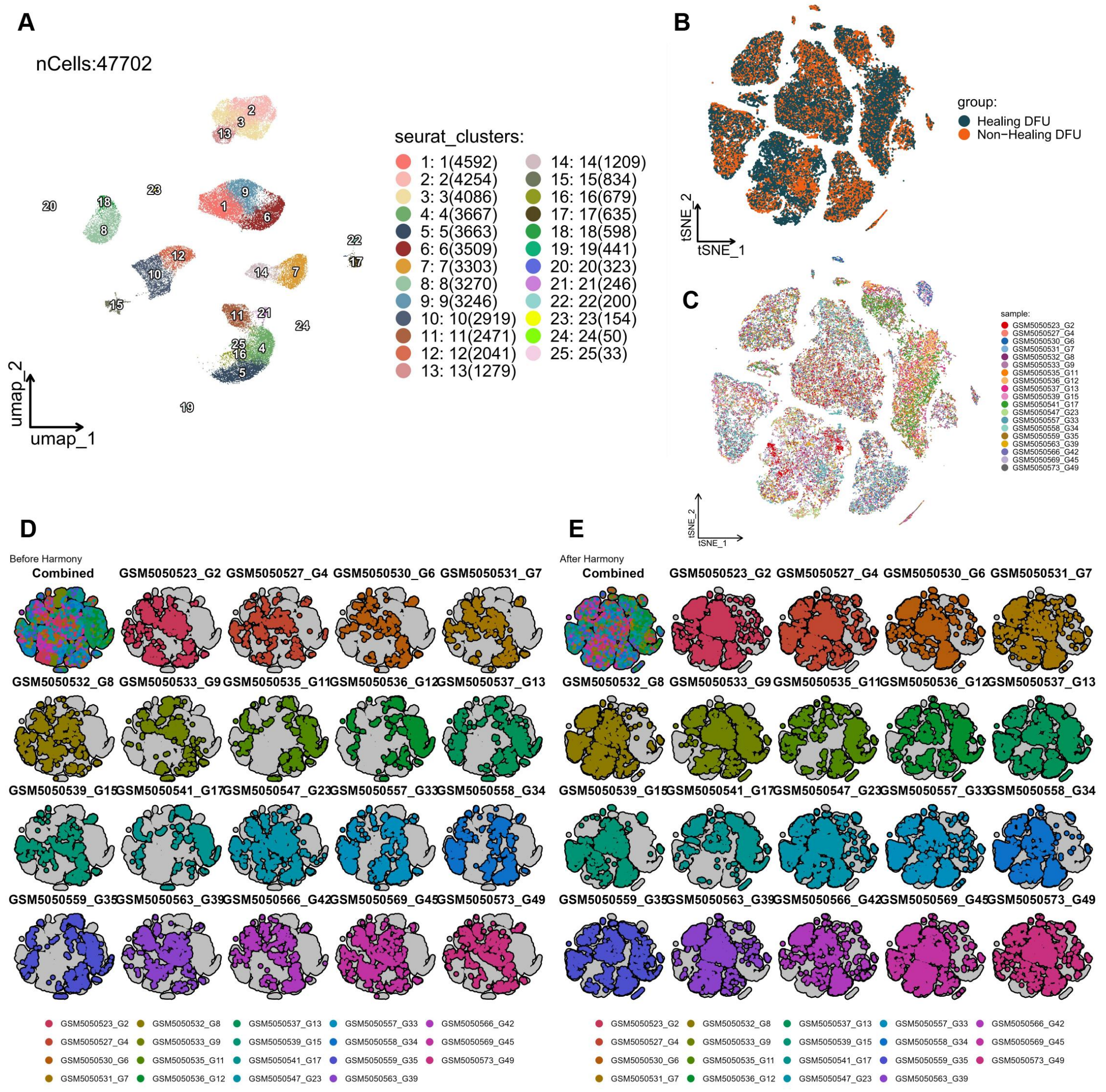

Supplementary Figure 2. *Through stringent quality control and unsupervised clustering analysis, cell clusters with similar expression patterns across diabetic wound samples were identified. (A-C) t-SNE visualization showing the distribution of cells by cluster, sample, and tissue origin. (D,E) t-SNE distribution of cells before and after batch effect removal using Harmony.*

Supplementary Table: *Highly expressed genes in various subpopulations of adipose-derived stem cells were screened using the FindAllMarkers function.*

p_val	avg_log2FC	pct.1	pct.2	p_val_adj	cluster	gene
3.57784848293857e-275	1.66267785767124	0.827	0.475	5.67732997272693e-271	1	C1QTNF3
1.12016095289989e-261	1.24320827917793	0.97	0.794	1.77747140006154e-257	1	PI16
3.73942845780671e-257	1.70685242810116	0.791	0.374	5.93372507684769e-253	1	PRG4
3.54742484454824e-197	1.10571359250779	0.944	0.819	5.62905374332915e-193	1	HTRA3
7.640195681484e-109	1.6298108330932	0.56	0.31	1.21234625073788e-104	1	FABP3
1.56588691432983e-100	1.33190573788903	0.49	0.234	2.48474935565857e-96	1	PMEPA1
2.8687591849446e-95	1.73654526124016	0.356	0.127	4.5521470746701e-91	1	TMEM35A
1.52858750350005e-85	1.12207186890879	0.514	0.275	2.42556265055388e-81	1	CD70
2.19354287751032e-80	1.20449958271525	0.584	0.364	3.48071383803337e-76	1	NR4A1
1.04489122850229e-71	1.5283815411352	0.416	0.211	1.65803340138743e-67	1	HAS1
5.06979597901229e-68	1.27300049750071	0.449	0.243	8.04475225949671e-64	1	PTHLH
1.26070446506465e-67	1.02973890783416	0.464	0.253	2.00048584516459e-63	1	BMP7
7.16127834544242e-46	1.00472575894244	0.324	0.166	1.1363516478548e-41	1	ADGRG2
5.891401246147e-36	1.00696716479404	0.311	0.176	9.34847549738606e-32	1	WNT10B
1.52444408814115e-31	1.01827058436817	0.278	0.154	2.41898787906238e-27	1	C6orf132
0	2.52979303859849	0.712	0.292	0	2	GPC3
3.87498937716528e-303	2.46864369171575	0.842	0.58	6.14883314368586e-299	2	APOD
3.36196036024039e-300	1.81785014468134	0.826	0.507	5.33475869962945e-296	2	CXCL12
5.81496059350087e-300	1.14756423941625	0.988	0.921	9.22717946976718e-296	2	COL6A3
4.772829716244	1.318540861	0.92	0.709	7.573526193736	2	OGN

04e-234	09146			04e-230		
3.7053632141913e-218	1.47839476328206	0.805	0.471	5.87967034827875e-214	2	ADH1B
2.20387265051207e-201	1.12782230629875	0.928	0.805	3.49710512183255e-197	2	CFH
6.65213378661416e-187	3.80809709910226	0.336	0.053	1.05556058925993e-182	2	MYOC
5.86743327978712e-176	1.85425902082458	0.562	0.241	9.31044312836621e-172	2	SRPX
3.03992846930114e-160	1.94445734534302	0.516	0.212	4.82375849508705e-156	2	FGL2
1.33985891413424e-133	2.04298706225415	0.441	0.171	2.12608812494821e-129	2	COL4A1
3.60903444692148e-124	1.41929457083119	0.593	0.341	5.726815860375e-120	2	SAMHD1
1.1238030014812e-114	1.86163406865928	0.391	0.147	1.78325060275037e-110	2	COL4A2
2.08619147147063e-103	1.10953897688159	0.762	0.573	3.3103686269296e-99	2	RND3
3.05497141158617e-99	1.97509150526787	0.353	0.134	4.84762863590493e-95	2	DHRS3
2.92721508560791e-97	1.63625450675881	0.392	0.162	4.64490489784264e-93	2	CYGB
4.08680106517669e-93	2.04716931239319	0.293	0.093	6.48493593022238e-89	2	FST
5.57538123401516e-86	2.48027534408418	0.348	0.141	8.84701494213526e-82	2	SFRP2
1.32722251995643e-82	1.06567954158655	0.593	0.384	2.10603669466687e-78	2	ABCA6
3.33692921073791e-82	1.63267285216593	0.363	0.159	5.29503927159891e-78	2	GAS6
6.24496738052169e-81	1.89722627672039	0.306	0.118	9.90951423941181e-77	2	BOC
8.33650125907321e-72	1.73590280004487	0.308	0.127	1.32283601978974e-67	2	CPB1
9.53519030842748e-72	1.57520026662716	0.364	0.176	1.51304399814127e-67	2	ANGPTL1
1.81384998342591e-71	1.08760961151919	0.557	0.374	2.87821715370024e-67	2	LAMA2
2.42260084745592e-69	1.02188604294534	0.586	0.401	3.84418302474306e-65	2	TMEM176B
6.03579610868138e-66	1.05669016931387	0.486	0.294	9.57760126525562e-62	2	PID1
2.355410511206	1.710417869	0.267	0.105	3.737565399181	2	MITF

03e-65	85623			73e-61		
6.26875152448184e-64	1.03244186135968	0.523	0.331	9.94725491904778e-60	2	THBS2
1.23539038663449e-57	2.21483480528257	0.344	0.184	1.96031746551161e-53	2	CXCL14
2.2711584989686e-56	1.03714574429933	0.469	0.293	3.60387430616337e-52	2	TMEM176A
4.61735184664794e-51	1.00712872656926	0.472	0.311	7.32681391026095e-47	2	COLEC12
1.23832634727033e-50	1.01074790487412	0.473	0.318	1.96497624784856e-46	2	FILIP1
1.35744708380721e-50	1.4588731114482	0.415	0.262	2.15399703258527e-46	2	IGFBP3
1.13496070572938e-49	1.36560786528619	0.312	0.16	1.80095564785139e-45	2	GDF10
1.22436150057121e-46	1.31705529017098	0.333	0.185	1.94281682910639e-42	2	ARRDC3
3.14425160723317e-46	1.28502534068274	0.303	0.158	4.98929845035759e-42	2	FAM13A
7.49968898643373e-46	1.04340078499771	0.367	0.211	1.1900506483673e-41	2	CPXM2
6.29946622036915e-45	1.24834783309142	0.323	0.179	9.99599299848176e-41	2	CPXM1
2.05534851124925e-44	1.31995609694703	0.342	0.197	3.26142701765032e-40	2	OGFRL1
6.25258101399676e-40	1.46281898117421	0.272	0.143	9.92159555301006e-36	2	MID1IP1
4.54653851598151e-39	1.13857356711169	0.303	0.169	7.21444731715946e-35	2	NEXN
4.53448527047147e-36	1.06980073340635	0.331	0.201	7.19532122718412e-32	2	LMO3
8.41127797557311e-34	1.06604478304863	0.426	0.305	1.33470158916394e-29	2	SOX4
6.79737784862368e-30	1.01518026166254	0.349	0.234	1.07860791701961e-25	2	FIBIN
8.58161145841572e-27	1.01526183648233	0.259	0.156	1.36173010622141e-22	2	ARHGAP18
1.2399109465949e-23	1.00353181757654	0.268	0.172	1.96749069005679e-19	2	CAMK2D
7.25231381459025e-74	4.03706218563983	0.432	0.071	1.15079715609918e-69	3	CCL2
1.18017097602309e-62	3.37569465733653	0.266	0.031	1.87269530475343e-58	3	BIRC3
7.818415787590	2.118928547	0.875	0.415	1.240626217174	3	IL32

61e-62	8667			88e-57		
1.154870435686<br>69e-54	2.883154785<br>75463	0.276	0.038	1.832548407347<br>64e-50	3	IL34
2.309226980745<br>8e-53	1.560303955<br>42525	0.927	0.532	3.664281373047<br>44e-49	3	TYMP
4.929486161408<br>75e-51	2.106149621<br>32269	0.802	0.337	7.822108640923<br>41e-47	3	PDPN
2.831284131841<br>05e-50	2.497271277<br>22779	0.354	0.065	4.492681660405<br>38e-46	3	NFKB2
3.186081014447<br>65e-48	1.931373873<br>22956	0.609	0.178	5.055673353725<br>53e-44	3	S1PR3
5.989261120808<br>67e-46	1.824600089<br>8947	0.74	0.31	9.503759546499<br>2e-42	3	PSME2
2.796746036720<br>85e-41	2.117315514<br>48799	0.292	0.052	4.437876611068<br>65e-37	3	RELB
1.310934230440<br>61e-38	1.688022315<br>17765	0.797	0.386	2.080190436863<br>17e-34	3	SOD2
3.996158303579<br>16e-37	1.222057266<br>68642	0.943	0.626	6.341103996119<br>42e-33	3	S100A1<br>6
1.834967002443<br>79e-36	1.159726881<br>82619	0.995	0.92	2.911725639477<br>8e-32	3	EMP3
2.177481980418<br>79e-34	1.641141795<br>3723	0.641	0.236	3.455228406528<br>54e-30	3	ADGRG2
3.292775276249<br>95e-34	1.775245308<br>49445	0.453	0.132	5.224975808353<br>43e-30	3	TRIM47
8.430051714340<br>5e-34	1.716514843<br>28057	0.526	0.177	1.337680606031<br>55e-29	3	MATN2
2.340197821716<br>53e-33	1.517658518<br>70949	0.677	0.29	3.713425903499<br>78e-29	3	TUBA1C
6.163042086676<br>99e-31	1.644769543<br>06659	0.594	0.24	9.779515183139<br>04e-27	3	NME1
1.047318163116<br>23e-29	2.057292323<br>0515	0.49	0.171	1.661884461232<br>83e-25	3	SLC39A<br>14
1.025072639035<br>36e-28	1.720005520<br>92434	0.552	0.22	1.626585263621<br>32e-24	3	IRF1
1.109130215298<br>94e-28	1.318004487<br>54151	0.615	0.258	1.759967825636<br>35e-24	3	TNFRSF<br>12A
4.706325122398<br>76e-28	1.687541243<br>35615	0.984	0.915	7.467996704222<br>35e-24	3	MT2A
5.573965038529<br>48e-28	1.013908483<br>90283	0.932	0.699	8.844767723138<br>58e-24	3	TPM4
1.313451369135<br>e-27	1.499750647<br>89318	0.589	0.247	2.084184632543<br>41e-23	3	SERPIN<br>E1
1.278325897775	1.503913601	0.479	0.165	2.028447534590	3	STING1

79e-26	09461			63e-22		
1.28560182848183e-26	1.23334339468239	0.635	0.267	2.03999298143496e-22	3	GASK1B
4.73219841915668e-26	1.57000412984472	0.422	0.141	7.50905245151783e-22	3	WNT4
1.3965895025545e-24	1.90486666562666	0.323	0.093	2.21610822265347e-20	3	CDKN2B
4.1966018072011e-24	1.67130480000175	0.312	0.088	6.65916774766671e-20	3	PAICS
1.60003707927645e-23	1.57268781453906	0.599	0.278	2.53893883739587e-19	3	TNFAIP3
1.81877512955109e-23	1.89785181824511	0.276	0.076	2.88603237557166e-19	3	PHLDA2
2.07601836651248e-22	1.02702370623382	0.828	0.486	3.294225943982e-18	3	HNRNPAB
3.53249209217762e-22	1.43176804118264	0.62	0.309	5.60535845186745e-18	3	HAS1
8.19874089321801e-21	1.74973536805725	0.323	0.102	1.30097620493583e-16	3	WARS1
1.21734539707517e-20	1.00996545240188	0.771	0.47	1.93168367607888e-16	3	CKAP4
1.95446127985173e-20	1.05518526011393	0.667	0.326	3.10133915886872e-16	3	ZNF385A
3.33083765569077e-20	1.4077119100795	0.401	0.148	5.28537319205011e-16	3	APOL6
4.24644032251594e-20	1.27601405068367	0.427	0.159	6.73825150376829e-16	3	PLEKHG4
5.68415819901957e-20	1.31047592162128	0.427	0.163	9.01962223020425e-16	3	CXXC5
6.04285870120084e-20	1.57651488010376	0.266	0.075	9.5888081870655e-16	3	NINJ1
2.55928346845079e-19	1.39701718029812	0.427	0.167	4.06107100773772e-15	3	APOL1
1.6901098595823e-18	1.55393593859492	0.911	0.761	2.68186632518519e-14	3	MT1E
1.74298397423794e-18	1.70538766934821	0.255	0.075	2.76576697032077e-14	3	GAP43
2.95168010204448e-18	1.33987623935464	0.391	0.149	4.68372598592419e-14	3	CTSS
4.43602773792662e-18	1.47928640111076	0.427	0.174	7.03908881454197e-14	3	OSMR
8.48938798630687e-18	1.25878160458304	0.318	0.106	1.34709608566717e-13	3	REL
7.518584627530	1.026658321	0.714	0.4	1.193049008696	3	DDX21

31e-17	72748			51e-12		
9.857793679184 87e-17	1.421635877 00084	0.635	0.355	1.564234701013 06e-12	3	NAMPT
1.087742848582 51e-16	1.275548514 2461	0.714	0.432	1.726030352130 72e-12	3	TNFAIP 6
2.040182415722 85e-16	1.079712686 46287	0.495	0.222	3.237361457269 02e-12	3	PSMB9
2.390346771682 94e-16	1.100731605 12286	0.411	0.164	3.793002257306 49e-12	3	DRAM1
2.868737752965 4e-16	1.152958132 25742	0.464	0.209	4.552113066405 5e-12	3	FLNB
3.101639819337 19e-16	1.460018190 59626	0.406	0.175	4.921682065324 25e-12	3	PIM3
1.175128085397 2e-15	1.243226523 12757	0.427	0.187	1.864693245908 27e-11	3	UGCG
1.488806341849 92e-15	1.269588400 6199	0.333	0.127	2.362437903247 45e-11	3	KDM6B
3.826539445572 36e-15	1.112864186 12908	0.734	0.476	6.071952792234 22e-11	3	SOCS3
5.104627065672 28e-15	1.167932388 64964	0.401	0.17	8.100022227808 77e-11	3	GUCY1A 2
6.051045702814 52e-15	1.161442458 4031	0.391	0.164	9.601799321226 09e-11	3	HSD11B 1
1.995101058325 87e-14	1.159682646 49632	0.333	0.131	3.165826359351 49e-10	3	ACKR2
9.358447383701 22e-14	1.412980219 55454	0.354	0.15	1.484998430845 71e-09	3	CFB
1.245999090301 52e-13	1.266164310 79212	0.271	0.098	1.977151356490 44e-09	3	ODC1
1.800982159063 87e-13	1.441908212 53866	0.292	0.111	2.857798490002 55e-09	3	NNT
1.548289595580 79e-12	1.439088093 44636	0.854	0.692	2.456825930267 59e-08	3	MT1X
2.032142220250 02e-12	1.089117473 19059	0.297	0.115	3.224603275092 72e-08	3	TP53
2.217602555847 12e-12	1.261058471 88979	0.339	0.144	3.518891735618 21e-08	3	RUNX1
3.212409205580 86e-12	1.272949124 53581	0.25	0.092	5.097450927415 71e-08	3	CCDC28 B
2.792692330028 3e-11	1.147082194 09126	0.307	0.132	4.431444189288 91e-07	3	TNFSF4
1.726938612813 73e-10	1.449632872 19041	0.828	0.661	2.740306190812 83e-06	3	SFRP4
2.038572564250	1.005079475	0.312	0.138	3.234806944952	3	MMP19

36e-10	26756			47e-06		
2.86912786944162e-10	1.11030683240299	0.417	0.211	4.55273210322997e-06	3	PLSCR1
4.12912066681019e-10	1.02656252246731	0.333	0.156	6.55208867409442e-06	3	EHD1
6.93530418297876e-10	1.06349625607923	0.255	0.106	1.10049406775507e-05	3	KCNK1
1.22180861787725e-09	3.50203862510413	0.255	0.115	1.93876591484761e-05	3	PTX3
2.71670937216628e-09	1.02218314926228	0.333	0.157	4.31087443175345e-05	3	STAT1
3.7026970713027e-09	1.01746184696708	0.339	0.172	5.87543971274312e-05	3	IER3
4.83664286158066e-09	1.04226880537104	0.359	0.185	7.67478489275619e-05	3	EDIL3
9.94837395660314e-09	1.58406975010955	0.484	0.3	0.000157860797943379	3	MT1A
3.30738131122467e-08	1.04255647903363	0.312	0.158	0.00052481526646513	3	SGK1
2.02106255070941e-06	1.22789275512088	0.755	0.621	0.0320702205546569	3	MT1M
2.47623993949971e-05	1.4315009769654	0.255	0.144	0.392929753599814	3	RARRES1
1.25150130844326e-174	6.03444660951501	0.729	0.032	1.98588227623776e-170	4	MX1
3.16339083199354e-124	5.25026269141876	0.593	0.03	5.01966857220735e-120	4	IFI44L
2.48864592361531e-121	5.16197600931644	0.492	0.02	3.94898335159277e-117	4	EPSTI1
4.3365049957976e-67	4.05063959019238	0.576	0.054	6.88116612733162e-63	4	OAS1
2.27021179877212e-64	4.91007800968204	0.847	0.147	3.60237208229161e-60	4	ISG15
5.11643638361855e-49	3.66839581666881	0.695	0.111	8.11876125352592e-45	4	MX2
7.91993223579471e-36	3.44490383350791	0.373	0.041	1.2567348471759e-31	4	DDX58
1.07389788381516e-33	3.12155636	0.695	0.157	1.70406116203789e-29	4	STAT1
1.78453824763983e-28	2.86487273687405	0.949	0.517	2.83170529135489e-24	4	IFI6
9.22206658880579e-28	2.91901511679686	0.559	0.116	1.4633575263117e-23	4	XAF1
2.038381996409	3.500603992	0.271	0.029	3.234504551901	4	SLC15A

e-26	36775			8e-22		3
9.34884273003779e-25	3.36615744541394	0.508	0.11	1.4834743644024e-20	4	IFIT3
1.71576443959756e-24	2.41251699210404	0.712	0.213	2.72257501275341e-20	4	PLSCR1
2.26850319366212e-24	3.20670164659384	0.458	0.087	3.59966086770306e-20	4	IFI44
1.10729326038118e-23	1.15893297606069	1	0.98	1.75705294557286e-19	4	IFITM3
5.81089321289556e-23	1.90724377926221	0.966	0.657	9.22072535022268e-19	4	LY6E
1.57767349668044e-21	2.81122215156697	0.746	0.276	2.50345230453252e-17	4	IFITM1
1.42235009831608e-19	2.67644583765696	0.288	0.042	2.25698513600795e-15	4	IRF9
2.47743786393898e-19	2.69626078473192	0.559	0.159	3.93119840249838e-15	4	IFIT1
5.91959079022757e-18	2.21051402143816	0.593	0.187	9.39320666593311e-14	4	IFI35
6.37371475796767e-17	2.34949348090186	0.356	0.07	1.01138105779431e-12	4	IRF7
8.49232182037499e-17	2.44539802379482	0.305	0.053	1.3475616264571e-12	4	HELZ2
2.31448229853209e-16	2.24639823670278	0.542	0.164	3.67262051131073e-12	4	PARP14
3.1306200822463e-14	1.68395952850453	0.966	0.844	4.96766794650843e-10	4	IFI27
6.56966481964267e-13	1.93525962809844	0.644	0.304	1.0424744135809e-08	4	EIF2AK2
1.15553453452743e-12	2.57985683282249	0.305	0.071	1.83360219938813e-08	4	TAP2
1.17211315881495e-11	1.72630648064742	0.542	0.201	1.85990916040756e-07	4	LAP3
6.88849380152805e-11	1.70238858416641	0.458	0.154	1.09306619642647e-06	4	SHFL
9.16144389054597e-11	1.75750967268498	0.559	0.228	1.45373791655183e-06	4	PSMB9
2.95513726646008e-10	1.37004748173955	0.831	0.542	4.68921181441885e-06	4	TYMP
1.18805762278898e-09	1.56368681641172	0.525	0.228	1.88520983584155e-05	4	ADAR
2.32931074333187e-09	1.47074015099065	0.678	0.383	3.69615028751901e-05	4	SP100
2.730765835445	2.137132778	0.288	0.08	4.333179227685	4	PARP10

86e-09	73505			5e-05		
3.041836657536<br>4e-09	1.668132165<br>98743	0.678	0.372	4.826786408178<br>76e-05	4	TNFSF1<br>0
6.105040766671<br>32e-09	1.544723759<br>1159	0.576	0.279	9.687478688554<br>05e-05	4	RNF213
1.343354563917<br>02e-08	1.390383667<br>10986	0.492	0.202	0.000213163502<br>202353	4	SP110
7.863271122178<br>22e-08	1.374004868<br>75877	0.508	0.228	0.001247743861<br>66724	4	STAT2
1.628524361710<br>53e-07	1.455309579<br>03879	0.322	0.108	0.002584142457<br>16226	4	GBP1
2.381930052553<br>79e-07	1.354428391<br>72135	0.525	0.25	0.003779646607<br>39235	4	TRIM22
3.187757612422<br>51e-07	1.666240137<br>17256	0.492	0.248	0.005058333779<br>39204	4	UBE2L6
1.234949887897<br>61e-05	1.471397609<br>28549	0.305	0.121	0.195961848211<br>593	4	ODF3B
1.409365864421<br>06e-05	1.099627635<br>58794	0.559	0.322	0.223638175366<br>335	4	PSME2
1.999674319736<br>01e-05	1.031474057<br>47804	0.525	0.288	0.317308321055<br>71	4	RBCK1
2.198345120927<br>91e-05	1.557642253<br>21221	0.254	0.094	0.348833403788<br>841	4	SAMD9L
3.130625412723<br>02e-05	1.778129634<br>26841	0.271	0.105	0.496767640490<br>888	4	SAMD9
9.240774360873<br>4e-05	1.301086819<br>84478	0.271	0.11	1	4	ANKFY1
0.000130990559<br>750483	1.029730187<br>00553	0.271	0.106	1	4	ALG14
0.000133241857<br>436739	1.194322219<br>9099	0.407	0.214	1	4	PHF11
0.000144121855<br>887315	1.262084084<br>46561	0.322	0.148	1	4	KPNA2
0.000147145746<br>229283	1.007189163<br>42934	0.322	0.143	1	4	WDR18
0.000258151686<br>903276	1.449424542<br>36512	0.254	0.11	1	4	OGFR
0.000277023046<br>112842	1.321890982<br>67064	0.322	0.154	1	4	APOL6
0.000283575539<br>984358	1.142971101<br>08223	0.356	0.175	1	4	ZHX1
0.000386790135<br>043768	1.291419460<br>50206	0.373	0.185	1	4	DDX60L
0.000895610415	1.033054743	0.356	0.188	1	4	SGMS1

242546	5754					
0.00164095765955467	1.10361654096404	0.288	0.144	1	4	CNDP2
0.00192319082506478	1.25025889653864	0.254	0.122	1	4	PNPT1
0.00203358980462528	1.36536742204616	0.254	0.122	1	4	ATP10D
0.00219046018841728	1.02389014397817	0.339	0.189	1	4	EDIL3
0.00318050153122245	1.24261980002373	0.254	0.127	1	4	AADACL2
0.00323248884200117	1.05330871229619	0.288	0.152	1	4	GTF2E2
0.00410929464348622	1.08870246192024	0.305	0.169	1	4	GPC6
0.00683371606857835	1.04122839881481	0.271	0.151	1	4	CARD6
0.00696918560767988	1.01745870253034	0.288	0.155	1	4	PI4KB
0.0075035569379202	1.00077061828891	0.271	0.146	1	4	CLIP4
0.00770737463429076	1.05136056618245	0.288	0.164	1	4	ZBED1
0.00892463852295998	1.02225390200359	0.254	0.135	1	4	C4orf48
